# GSTCD and INTS12 Regulation and Expression in the Human Lung

**DOI:** 10.1371/journal.pone.0074630

**Published:** 2013-09-18

**Authors:** Ma’en Obeidat, Suzanne Miller, Kelly Probert, Charlotte K. Billington, Amanda P. Henry, Emily Hodge, Carl P. Nelson, Ceri E. Stewart, Caroline Swan, Louise V. Wain, María Soler Artigas, Erik Melén, Kevin Ushey, Ke Hao, Maxime Lamontagne, Yohan Bossé, Dirkje S. Postma, Martin D. Tobin, Ian Sayers, Ian P. Hall

**Affiliations:** 1 Division of Respiratory Medicine, University of Nottingham, Queen’s Medical Center, Nottingham, United Kingdom; 2 Genetic Epidemiology Group, Department of Health Sciences, University of Leicester, Leicester, United Kingdom; 3 Institute of Environmental Medicine, Karolinska Institutet and Sachs’ Children’s Hospital, Stockholm, Sweden; 4 James Hogg Research Centre, Institute for Heart and Lung Health, University of British Columbia, Vancouver, British Columbia, Canada; 5 Department of Genetics and Genomic Sciences, Icahn Institute of Genomics and Multiscale Biology, Icahn School of Medicine at Mount Sinai, New York, United States of America; 6 Department of Molecular Medicine, Laval University, Québec City, Canada; 7 Institut Universitaire de Cardiologie et de Pneumologie de Québec, Laval University, Québec City, Canada; 8 Department of Pulmonology, University of Groningen, University Medical Center Groningen, Groningen, The Netherlands; 9 National Institute for Health Research (NIHR) Leicester Respiratory Biomedical Research Unit, Glenfield Hospital, Leicester, United Kingdom; University of Giessen Lung Center, Germany

## Abstract

Genome-Wide Association Study (GWAS) meta-analyses have identified a strong association signal for lung function, which maps to a region on 4q24 containing two oppositely transcribed genes: glutathione S-transferase, C-terminal domain containing (*GSTCD*) and integrator complex subunit 12 (*INTS12*). Both genes were found to be expressed in a range of human airway cell types. The promoter regions and transcription start sites were determined in mRNA from human lung and a novel splice variant was identified for each gene. We obtained the following evidence for *GSTCD* and *INTS12* co-regulation and expression: (i) correlated mRNA expression was observed both *via* Q-PCR and in a lung expression quantitative trait loci (eQTL) study, (ii) induction of both *GSTCD* and *INTS12* mRNA expression in human airway smooth muscle cells was seen in response to TGFβ1, (iii) a lung eQTL study revealed that both *GSTCD* and *INTS12* mRNA levels positively correlate with percent predicted FEV_1_, and (iv) FEV_1_ GWAS associated SNPs in 4q24 were found to act as an eQTL for *INTS12* in a number of tissues. In fixed sections of human lung tissue, GSTCD protein expression was ubiquitous, whereas INTS12 expression was predominantly in epithelial cells and pneumocytes. During human fetal lung development, GSTCD protein expression was observed to be highest at the earlier pseudoglandular stage (10-12 weeks) compared with the later canalicular stage (17-19 weeks), whereas INTS12 expression levels did not alter throughout these stages. Knowledge of the transcriptional and translational regulation and expression of *GSTCD* and *INTS12* provides important insights into the potential role of these genes in determining lung function. Future work is warranted to fully define the functions of *INTS12* and *GSTCD*.

## Introduction

Forced expiratory volume in one second (FEV_1_), forced vital capacity (FVC), and the ratio of FEV_1_ to FVC (FEV _1_/FVC) are commonly used to assess pulmonary function and these measurements are integral to the diagnosis of chronic obstructive pulmonary disease (COPD). Reduced FEV _1_/FVC defines airway obstruction, whereas reduced FEV_1_ grades the severity of airway obstruction [1]. These measures also predict population morbidity and mortality [2].

Pulmonary function is determined both by environmental and genetic factors. Tobacco smoking is the major environmental risk factor for the development of COPD in the developed world. A genetic contribution to pulmonary function is well established with heritability estimates reaching as high as 77% for FEV_1_ [3]. Three large scale meta-analyses of genome-wide association studies (GWAS) of lung function measures have recently been published [4-6]: these studies identified a total of 26 novel loci associated with either FEV_1_ or FEV _1_/FVC. Genome-wide interaction analyses with smoking later identified three additional regions of potential importance for lung function [7]. One of the strongest association signals identified was with intronic Single Nucleotide Polymorphisms (SNPs) in a region at 4q24 containing two oppositely transcribed genes: glutathione S-transferase, C-terminal domain containing (*GSTCD*) and integrator complex subunit 12 (*INTS12*). In the SpiroMeta study [5], SNP rs10516526 in intron 5 of *GSTCD* was associated with FEV_1_ (*P*=2.18x10^-23^ in the joint meta-analysis of discovery and replication cohorts (n=53,309)). In the combined SpiroMeta CHARGE meta-analysis with larger sample size (discovery n=48,201), SNP rs10516526 also showed the strongest association in the 4q24 locus for FEV_1_ (*P*=4.75x10^-14^) [6]. Subsequent reports by SpiroMeta investigators and others have also implicated this locus for association with COPD [8,9].

At present, little is known about the potential function of GSTCD – all four entries on PubMed are related to recent GWAS studies [4,5,8,9]. GSTCD is so named because of homology with the glutathione S-transferase (GST) super family of enzymes [10]. GSTs are also involved in the detoxification of products of oxidative stress [11] and synthesis of steroid hormones. They also have an increasingly appreciated role in cell signalling pathways including regulation of Jun N-terminal kinase and the modulation of ryanodine receptor calcium channels [10,12]. However, GSTCD lacks key functional domains important for GST activity. Although still only appearing in a handful of (largely GWAS-related) publications [4,8,13], more is known about the function of INTS12, which is a subunit of the Integrator complex. This complex associates with the C-terminal domain of RNA polymerase II large subunit and mediates 3` end processing of small nuclear RNAs [14].

Additional evidence linking *GSTCD* to variation in lung function came from a study reporting human lung mRNA correlation with lung function [15]. In this study, which undertook genome-wide expression profiling, *GSTCD* mRNA levels were associated with percent predicted FEV_1_ (Spearman correlation=0.44, *P*=0.001) and the FEV _1_/FVC ratio (Spearman correlation=0.48, *P*=0.0002) [15]. This, and the fact that the 4q24 locus has the strongest novel association with lung function in the multiple GWAS meta-analyses conducted so far, have provided the rationale to further investigate this locus to unravel the mechanisms underlying this association.

Here we report the genetic architecture of *GSTCD* and *INTS12*, and show that both genes are expressed in a range of cell types present in the lung. We report one novel splice variant for each gene. Furthermore we show that the genome-wide significant SNPs at 4q24 act as expression quantitative trait loci (eQTL) for *INTS12*, and provide evidence that the peak association signal maps to a region rich in elements likely to be important for transcriptional regulation of these genes. Interestingly, these appear to be coordinately regulated in the lung. Expression levels were also shown to correlate with lung function measures. Finally, we describe the expression of GSTCD and INTS12 proteins in human lung tissue and assess how expression is altered during human fetal development.

## Results

### Gene arrangement *via* PCR and RACE

**Figure 1:**
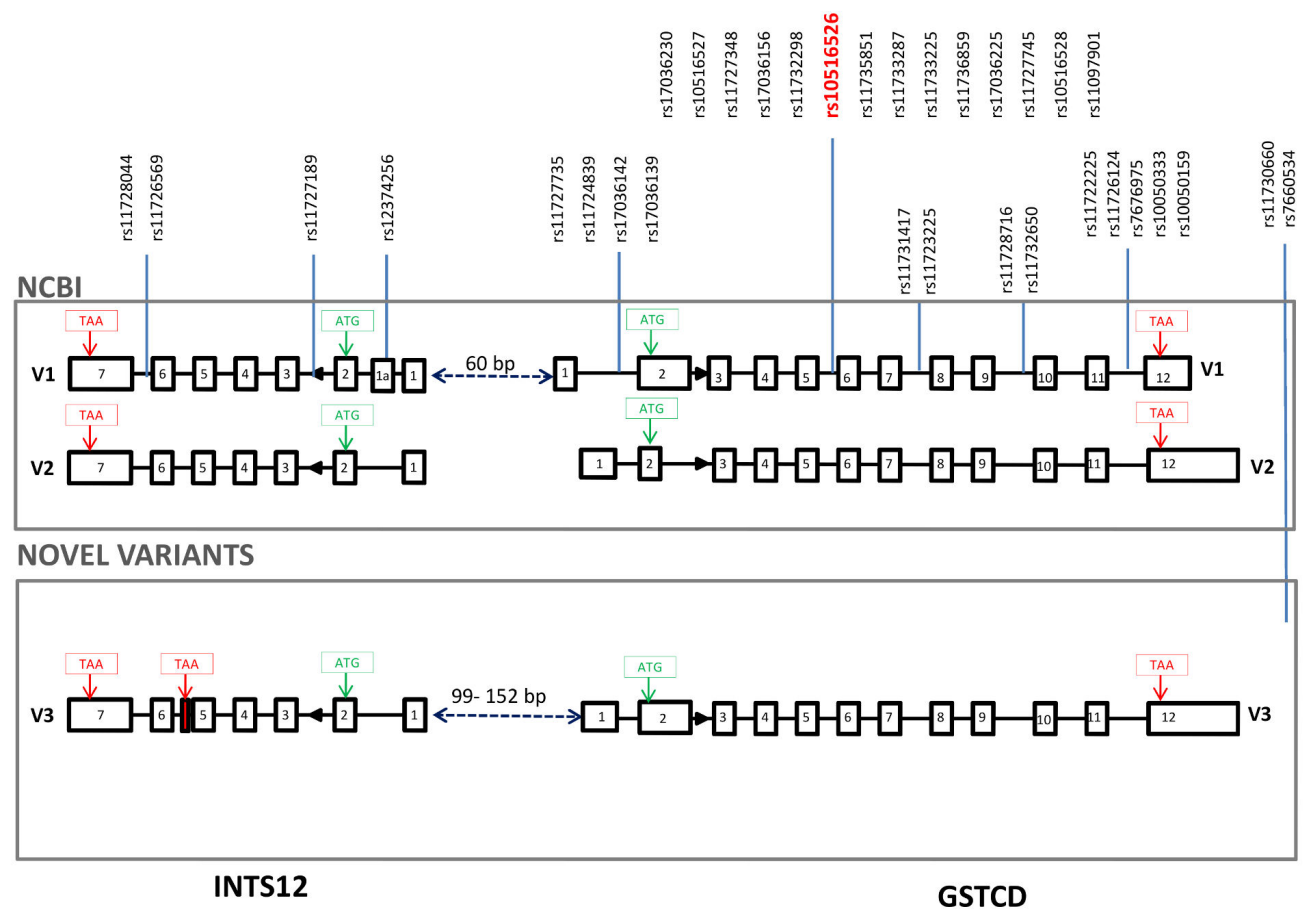
Genetic architecture of the region containing both *GSTCD* and *INTS12* genes. The top panel depicts gene arrangements previously reported in NCBI, build 37, whereas the lower panel shows novel variants identified in lung. V1, 2, 3 refer to splice variants 1, 2 and 3 for each gene. Open boxes represent exons and connecting black lines represent introns. Also illustrated are the locations of Single Nucleotide Polymorphisms (SNPs) meeting genome-wide association (*P*≤5x10^-8^) for FEV_1_ in previously reported analyses of the SpiroMeta consortium [5]. Highlighted in red is the sentinel SNP rs10516526 that was associated with FEV_1_ (*P*=2.18 x 10^-23^ in all stage analyses) [5]. Translation start codons (ATG) are shown boxed in green and stop codons (TAA) boxed in red.

Using cDNA synthesised from RNA extracted from total lung, human airway smooth muscle (HASM) cells and normal human bronchial epithelial cells (HBEC), Reverse Transcription (RT)-PCR was initially performed to investigate whether gene arrangements are consistent with those reported by NCBI (the National Center for Biotechnology Information). For both *GSTCD* and *INTS12* a novel splice variant was discovered *via* this method (named here “variant 3”). Figure 1 outlines schematically the two published variants in addition to the novel variant 3 in each case. The SpiroMeta genome-wide significant SNPs for association with FEV_1_ in this locus are annotated on the schematic in Figure 1 along with the relevant association statistical *P* values [5].

To confirm published or reveal novel transcription start site (TSS) s for both *GSTCD* and *INTS12*, Rapid Amplification of cDNA Ends (RACE) was performed. For *GSTCD*, a total of 30 clones were sequenced and analysed. 5’ RACE confirmed the presence of variant 2 (NM_024751.2) in 13 of these (43%), albeit with multiple TSSs spanning a region of 50bp; minimum +3, maximum +53bp distance relative to RefSeq (TSS) at 106629941 (hg build 37) (Figure S1A). The annotated nucleotide sequence showing TSSs in both *GSTCD* and *INTS12* can be found in Figure S1. Interestingly, variant 1 of *GSTCD* (NM_001031720.2) was not identified in any of the 30 RACE clones analysed. Sequence reads did confirm the novel variant 3 identified *via* RT-PCR for *GSTCD* in 17 clones out of the 30 analysed (56%) (Figure S1B). Variant 3 sequence mapped to the first exon of variant 2 and the second exon of variant 1. Three different TSSs were identified in variant 3 spanning a region of 27bp; the first was identical to the RefSeq exon 1 (3/30 clones = 10%), the second was the most common (12/30 clones = 40%) and had a 10bp shorter exon 1, the third and least frequent had a 27bp shorter exon 1 (2/30 clones= 6.7%) (Figure S1A and B).

To confirm the absence of exon 1 of *GSTCD* variant 1 in the lung tissue, a different RT-PCR protocol was designed with primers spanning this exon and a sequence in exon 3, common to both variants. No PCR product for this assay was detected in a panel of airway-related cell types after 40 cycles of PCR (Figure S2).

For *INTS12*, the 5’ RACE using RNA from lung tissue confirmed the presence of variant 2 with multiple TSSs over a region of ~30bp; minimum 36 and maximum 60bp upstream (distance relative to the RefSeq build 37 TSS at 106629881). Variant 1 (NM_020395.3) was not observed in any of the 31 clones analysed. The most common variant 2 transcript identified was a 60bp truncated first exon (18/31 clones = 58%), relative to the RefSeq. 50bp and 36bp truncations of exon 1 were also identified with frequencies of 23 and 19%, respectively (Figure S1C).

As shown in Table 1, both *GSTCD* and *INTS12* feature on NCBI as existing in two variants. *GSTCD* variants 1 and 2 encode two proteins differing in length by 87 amino acids. This is the result of a truncated second exon meaning that amino acids 55 to 141 of variant 1 are absent in variant 2. Unlike *GSTCD*, the two *INTS12* transcripts both encode the same protein. If translated, the *INTS12* variant 3 would result in a truncated protein of 237 residues (*versus* 462 residues for variants 1 and 2), due to a premature translational stop codon present in the additional exon between exons 5 and 6 (shown boxed in red in Figure 1). The resulting protein isoform retains the PHD-type zinc finger, but lacks the serine-rich region. Searches to identify proteins sharing homology with the GSTCD and INTS12 variants are shown in Table S1 (A-C) and summarised in the discussion.

**Table 1:** Protein variants of GSTCD and INTS12, corresponding mRNAs.

**Gene**	**Variant**	**mRNA**	**Protein**	**Amino acid length**
GSTCD	1	NM_001031720.3	NP_001026890.2	633
	2	NM_024751.3	NP_079027.2	546
	3	NK	NK	633
INTS12	1	NM_020395.3	NP_065128	462*
	2	NM_001142471.1	NP_001135943	
	3	NK	NK	237

Based on NCBI gene database accessed November 2012. NK: Not Known; * Both variants encode the same protein.

### Re-sequencing of the *GSTCD*/*INTS12* region

In two separate studies (results currently unpublished), re-sequencing experiments were performed to investigate the effects of novel or rare SNPs on lung function. We extracted information on SNPs measured in these studies within *GSTCD* and *INTS12* to identify whether there were any SNPs which were novel or not included in the previously used (i.e. HapMap) or next generation (i.e. 1000 Genomes Project) SNP imputation reference panels used for genome-wide association studies of the genetic determinants of lung function. An exome re-sequencing project using DNA from 100 individuals with normal FEV_1_ despite many years of heavy smoking (see Table S2A) identified 1 non-synonymous SNP (rs146172950) and 1 synonymous SNP (rs144650579) in the coding region of *INTS12* which were listed in dbSNP v137 but which were not in the 1000 Genomes Project imputation reference panel [16]. All SNPs appeared in the 1000 Genomes Project imputation reference panel in exonic *GSTCD*. In addition a targeted sequencing study in both controls and subjects with COPD that focused on 26 regions known to affect lung function [4,5,9] (see Table S2B) identified two intronic SNPs in *GSTCD* that do not appear in the 1000 Genomes Project imputation reference panel. One of them is listed in dbSNP v137 (rs113987854) and the other is not. None of these SNPs were in the widely used HapMap imputation panels and so would not have been studied in imputed genome-wide association studies undertaken to date. These data provide some evidence for variation in *GSTCD* that is not currently included in the next generation of widely used imputation reference panels of SNPs.

### *GSTCD*/*INTS12* in the University of California Santa Cruz (UCSC) Genome Browser

**Figure 2:**
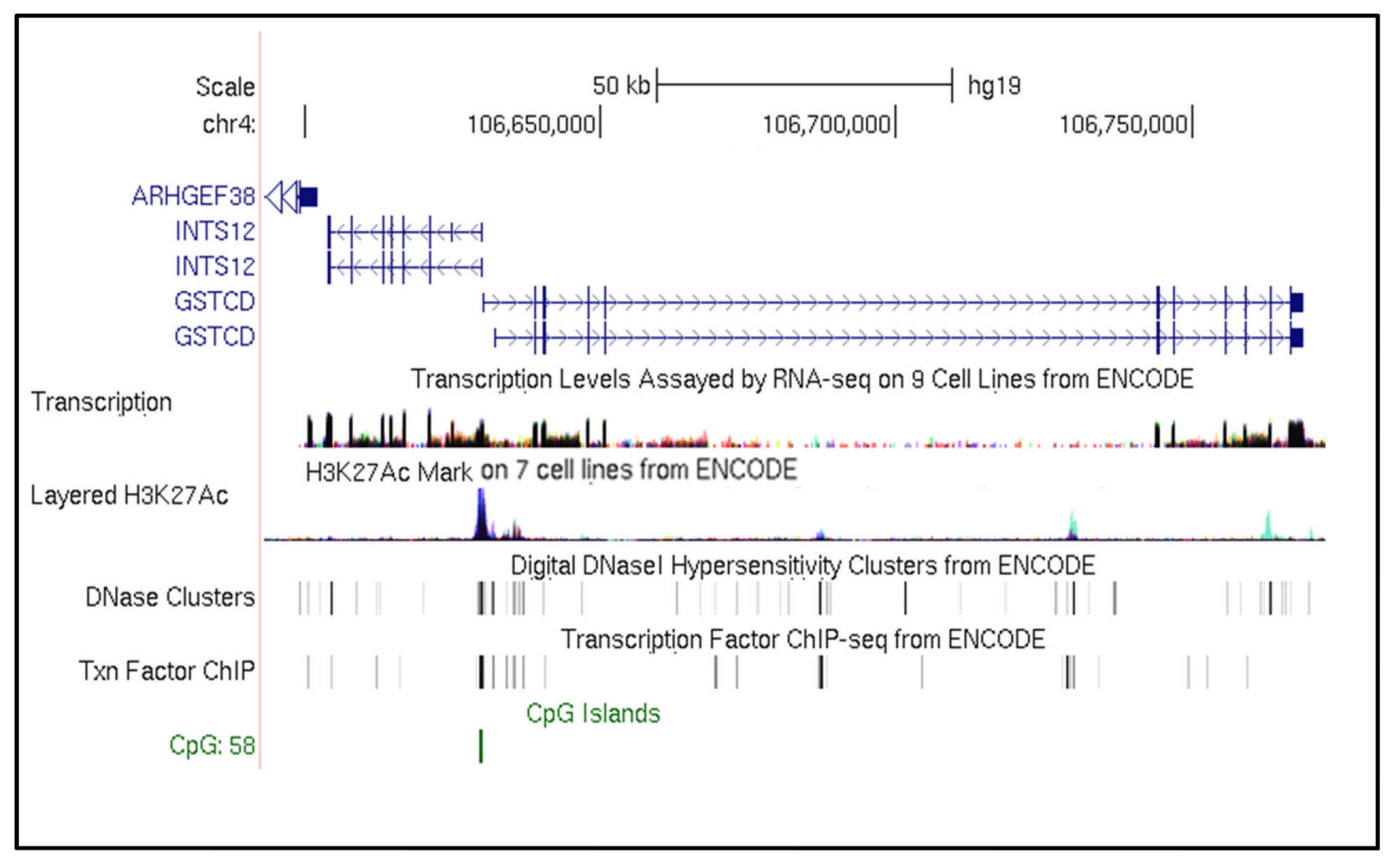
Regulatory motifs within the *GSTCD/INTS12* locus. The *GSTCD*/*INTS12* locus is shown, annotated with RNA sequencing, H3K27Ac histone marks, DNase hypersensitivity, transcription factor binding and CpG islands (UCSC Genome Browser (http://genome.ucsc.edu/)) on the Human Feb 2009 (GRCh37/hg19) assembly. For the H3K27Ac histone marks and RNA sequence tracks, peak height is proportional to signal amplitude, with colours representing datasets in different cell backgrounds (pale blue H3K27Ac histone trace = human umbilical vein endothelial cell (HUVEC); blue/grey = K562 erythroleukaemia cells). For the DNase hypersensitivity and transcription factor binding tracks, a grey band indicates the extent of the hypersensitive region and the intensity of the band is proportional to the maximum signal strength observed in any cell line.

Due to the close proximity of the *GSTCD* and *INTS12* genes, we investigated current knowledge regarding the regulation of this region through bioinformatic searches including data and tracks generated from the ENCODE project [17]. Figure 2 shows the “Regulation” tracks from the UCSC Genome Browser (http://genome.ucsc.edu/). It is interesting to note the histone modification activity in this region is expressed as acetylation of lysine 27 of the H3 histone protein; this is usually associated with enhanced transcription and active promoters in mammalian cells [18]. Also shown in Figure 2 is a CpG island (bottom track). An expansion of this region reveals the CpG island to be 639bp long: this encompasses the intergenic region between *GSTCD* and *INTS12*, and parts of the first exon of both genes. The UCSC Genome Browser defines a CpG island as a region of at least 200bp, with a GC percentage >50%, and an observed/expected CpG ratio >60%. CpG islands are classically associated with transcription start sites and are found in ~70% of annotated promoters [19].

### Expression of GSTCD and *INTS12* mRNA in human airway cells and lung tissue

**Figure 3:**
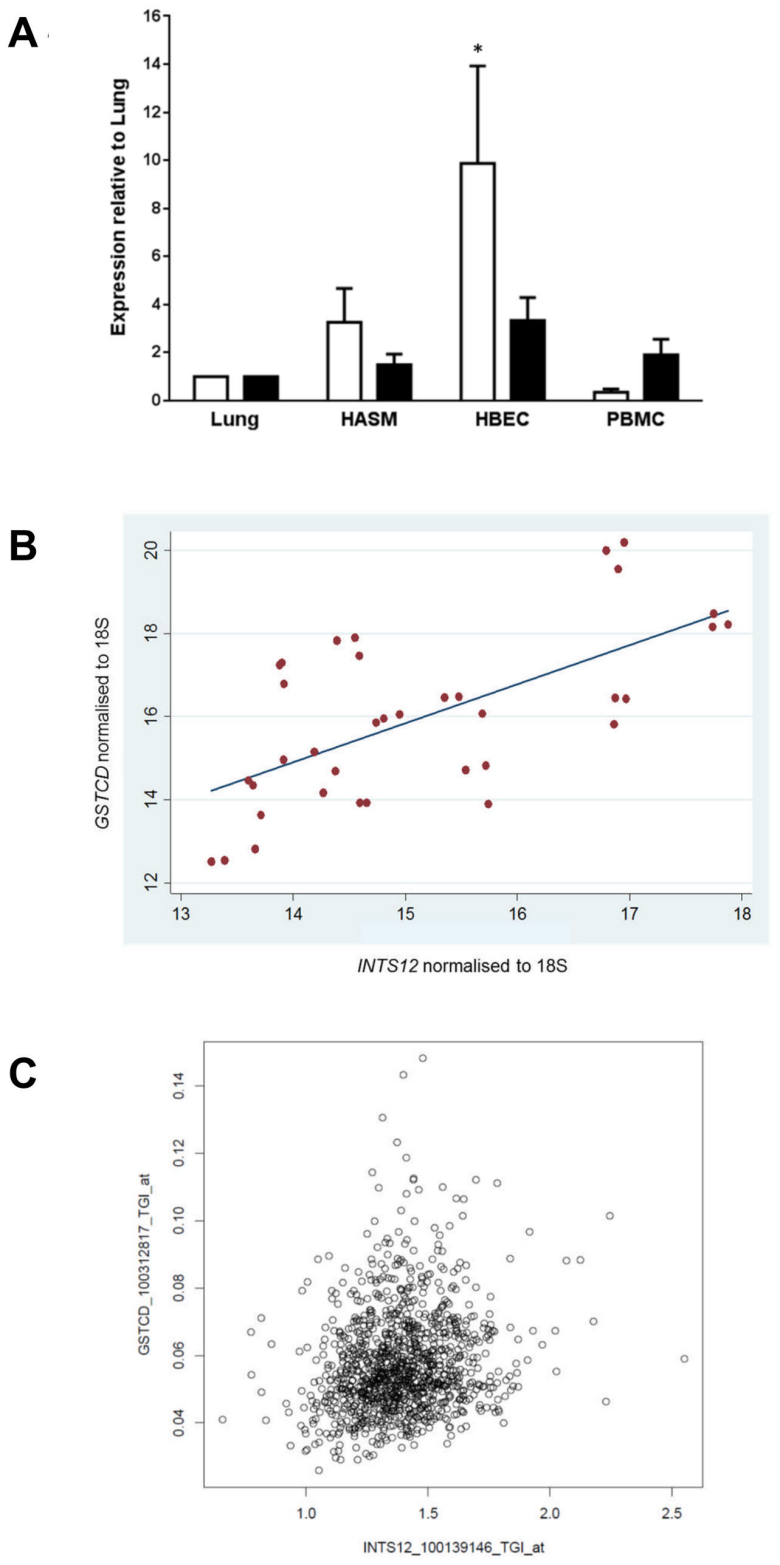
A: Expression of *GSTCD* and *INTS12* mRNA in Lung and Airway cells. mRNA expression in human airway smooth muscle (HASM) cells, human bronchial epithelial cells (HBEC) and peripheral blood mononuclear cells (PBMC) is shown relative to mRNA from lung. Open bars depict *GSTCD* expression whereas black bars show *INTS12* expression. Values shown are mean and standard error of the mean (SEM) (n=3). Only the expression of *GSTCD* in HBEC relative to lung was statistically significant (* *P*=0.0494). **B**: **Correlation between *GSTCD* and *INTS12* ΔCt values in HASM, HBEC, PBMC and lung**. mRNA expression levels as shown in Figure 3A from human airway smooth muscle (HASM) cells, human bronchial epithelial cells (HBEC), peripheral blood mononuclear cells (PBMC) and lung were correlated using a scatter plot. The correlation coefficient between these measures was r=0.8, *P*<0.0001. **C**: **Correlation between *GSTCD* and *INTS12* mRNA levels in the lung**. The scatter plot shows a positive correlation between the *GSTCD* and *INTS12* probe sets as investigated in the lung eQTL study [20].

We have previously reported that *GSTCD* is identified in lung and airway-related cell types at the mRNA level using RT-PCR [5], however the presence of *INTS12* was not assessed. The relative expression of *GSTCD* and *INTS12* was therefore determined in a panel of airway-related cell types using quantitative RT-PCR (Q-PCR). Whole human lung, cultured HASM cells, cultured HBEC and peripheral blood mononuclear cells (PBMC) were studied (Figure 3A). Mean expression data are presented relative to the whole lung sample for each tissue/cell type. The highest expression of both *GSTCD* and *INTS12* was seen in HBEC, with *GSTCD* showing relatively higher expression in this cell type than *INTS12*. The lowest *GSTCD* expression was detected in PBMC, while for *INTS12* the lowest expression was detected in the whole lung samples. Ct values observed for *GSTCD* and *INTS12* were generally comparable, and the efficiency standard curves for both genes were also similar (data not shown). Interestingly, delta Ct values of both genes presented as a scatter plot (Figure 3B) showed a moderate degree of correlation (r=0.64, *P*<0.0001) suggesting expression may be coordinately regulated. To verify this, we investigated the correlation between the *GSTCD* and *INTS12* probe sets in 1,111 human lung specimens from the lung eQTL study [20]. The results presented in Table 2 show good positive correlation between the *GSTCD* and *INTS12* probe sets in agreement with that observed *via* Q-PCR. The strongest correlation observed was between probe set 100139146_TGI_at (*INTS12*) and probe set 100312817_TGI_at (*GSTCD*) (r=0.18, *P*=6.7E-10); a scatter plot of these data is shown in Figure 3C.

**Table 2:** Correlation coefficients and *P* values for *GSTCD* and *INTS12* probe sets in 1,111 individuals from the lung eQTL study.

**GSTCD probe sets**	**INTS12_100139146_TGI_at**	
	**Pearson correlation**	**Pearson *P* value**
GSTCD_100156283_TGI_at	0.145	1.29E-06
GSTCD_100147268_TGI_at	-0.058	0.05151
GSTCD_100312817_TGI_at	0.184	6.67E-10

### Altered expression of GSTCD and *INTS12* mRNA by TGFβ1

**Figure 4:**
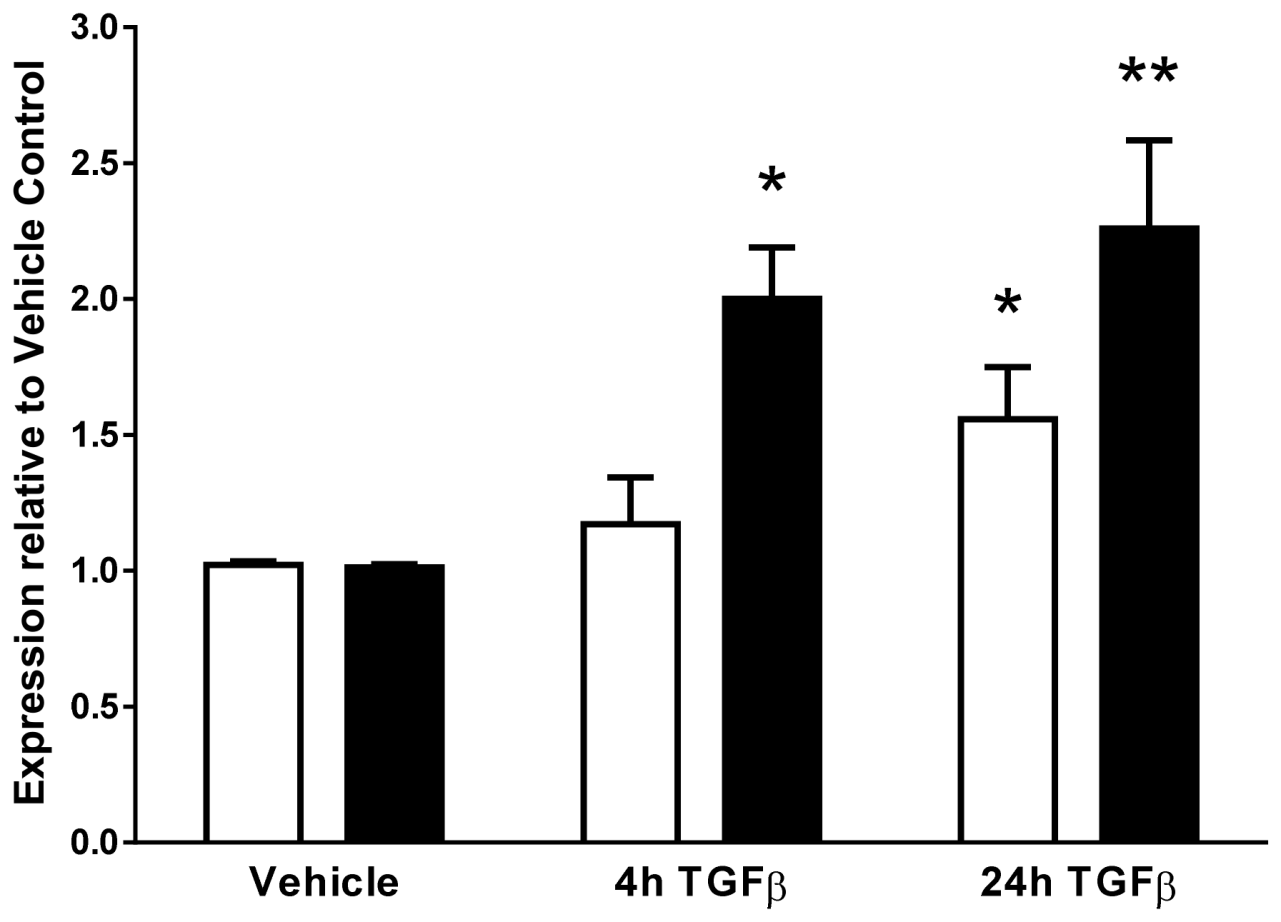
*GSTCD* and *INTS12* gene expression is altered following exposure of HASM cells to TGFβ1. Human airway smooth muscle (HASM) cells were exposed to 10ng/ml TGFβ1 for 4 or 24 hours. Open bars depict *GSTCD* expression whereas black bars show *INTS12* expression. Values shown are mean and standard error of the mean (SEM) (n=5). Significant increases in both *GSTCD* and *INTS12* gene expression were observed following 24h exposure to TGFβ1 (*P*<0.05 *GSTCD*, *P*<0.01 *INTS12*) and after 4h TGFβ1 exposure in *INTS12* expression (*P*<0.05).

Further evidence of coordinated expression came from a series of experiments to determine which signalling pathways could modulate mRNA levels of *GSTCD* and *INTS12* in HASM and HBEC, as assessed by Q-PCR. A range of agents was selected, each known to mediate common cell signalling pathways, particularly with a focus on those associated with airway dysfunction. The agents utilised were bradykinin (elevator of intracellular Ca^2+^), forskolin (activator of adenylyl cyclase and hence the pro-relaxant cyclic AMP pathway), lipopolysaccharide (LPS) (used as a mimic of bacterial infection), transforming growth factor β1 (TGFβ1) (a key cytokine, found to be elevated in airway inflammation, and in COPD) and the Extracellular Signal-Regulated Kinase (ERK) inhibitor U0126. None of the agents induced a significant alteration in the levels of mRNA expression of either gene in HBEC. In addition, of all the panel of reagents utilised, only exposure to TGFβ1 induced a significant change in mRNA expression in HASM cells, however this was observed for both genes (Figure 4). Significant increases in mRNA expression in HASM cells were observed following 24h exposure to TGFβ1 in both *GSTCD* (1.56 ± 0.2 fold cf vehicle control, n=5, *P*<0.05) and *INTS12* (2.26 ± 0.3 fold cf vehicle control, n=5, *P*<0.01) while a significant increase in *INTS12* was also observed following 4h TGFβ1 exposure (2.0 ± 0.2 fold cf vehicle control, n=5, *P*<0.05). To investigate the possible transcriptional mechanisms driving these observed differences, ENCODE data from the UCSC Genome Browser were utilised to investigate transcription factor binding as determined through ChIP-seq (chromatin immunoprecipitation with antibodies specific to the transcription factor followed by sequencing of the precipitated DNA). A number of transcription factor binding sites were identified in the intergenic region between *GSTCD* and *INTS12* and in the gene 5’ regions (Table S3) and the potential relevance of these is covered in the Discussion.

### Identifying eQTL in the *GSTCD/INTS12* locus

To gain further insight into the mechanisms underlying the GWAS signal on 4q24, we investigated the presence of eQTL in this locus using publically available eQTL sources, and also through access to a lung tissue-specific eQTL dataset in collaboration with the lung eQTL study group [20].

We investigated eQTL in the 4q24 locus *via* the eQTL website at http://www.hsph.harvard.edu/liming-liang/software/eqtl/ using the lymphoblastoid cell line dataset from Liang et al. [21]. Interestingly, using this dataset we identified an eQTL for *INTS12* that met the 0.05 false discovery rate (FDR) (probe set 218616_at). There were no significant eQTL for *GSTCD*, *NPNT* or *FLJ20184* (Figure S3A).

In addition, we investigated eQTL in the 4q24 region using the University of Chicago eQTL website at http://eqtl.uchicago.edu/Home.html which hosts a browser summarising results from a collection of published eQTL studies. The results shown in Figure S3B, are similar to the Liang et al., data suggesting the presence of eQTL for *INTS12* in eQTL studies utilising different tissue types. We then extracted the associations of *INTS12* eQTL with FEV_1_ in the SpiroMeta dataset, and interestingly the majority of *INTS12* eQTL show genome-wide significant associations with FEV_1_ in the SpiroMeta dataset. The *INTS12* eQTL and their associations with FEV_1_ are shown in Table 3. Additionally, we checked the potential function of *INTS12* eQTL in the ENCODE dataset using HaploReg v2. These eQTL appear to span regions with either known protein binding motifs, enhancers, promoters or DNase hypersensitivity sites. Results are shown in Table S4. In collaboration with the lung eQTL study [20] we aimed to identify eQTL for *GSTCD* and *INTS12* in the human lung using a dataset of genome-wide genotypes and gene expression profiles from the lungs of 1,111 individuals that has recently been published [20]. There were no lung eQTL for *GSTCD* or *INTS12* that pass the 0.1 FDR threshold set in the lung eQTL study. The eQTL identified for *GSTCD* and *INTS12* in the lung are shown in Figure S4 which suggest a stronger eQTL signal for *INTS12*, albeit not significant when correcting for multiple comparisons.

**Table 3:** *INTS12* eQTL identified in multiple tissue types and their association with FEV_1_ in the SpiroMeta study. LCL: lymphoblastoid cell lines.

**SNP**	**Position**	**FEV_1_*P* value**	**eQTL *P* value or significance**	**Tissue**	**Reference**
rs10050159	106985364	7.23E-09	6.59E-08	LCL	[21]
rs10050333	106985262	7.22E-09	6.55E-08	LCL	
rs10516525	106887474	1.44E-09	9.96E-08	LCL	
rs10516525	106887474	1.44E-09	0.014 (posterior probability)	LCL	[40]
rs10516525	106887474	1.44E-09	1.30E-07	Liver	[41]
rs10516526	106908353	6.67E-10	9.94E-08	LCL	[21]
rs10516527	106953795	6.49E-09	4.66E-08	LCL	
rs10516528	106959042	6.27E-09	6.48E-08	LCL	
rs11097901	106949382	6.32E-09	4.64E-08	LCL	
rs11722225	106985879	7.08E-09	6.60E-08	LCL	
rs11723225	106965514	6.02E-09	6.37E-08	LCL	
rs11724839	106857705	1.79E-09	9.94E-08	LCL	
rs11724839	106857705	1.79E-09	0.036 (posterior probability)	LCL	[40]
rs11726124	106985945	6.63E-09	6.63E-08	LCL	[21]
rs11726569	106826057	1.95E-09	0.075 (posterior probability)	LCL	[40]
rs11727189	106838589	3.38E-09	1.03E-07	LCL	[21]
rs11727735	106851319	2.10E-09	9.94E-08	LCL	
rs11727735	106851319	2.10E-09	0.14 (posterior probability)	LCL	[40]
rs11727745	106935976	5.47E-09	5.27E-08	LCL	[21]
rs11728044	106824235	1.95E-09	1.05E-07	LCL	
rs11728044	106824235	1.95E-09	0.185 (posterior probability)	LCL	[40]
rs11728716	106975445	8.44E-09	1.28E-07	Cortex (Brain)	[42]
rs11731417	106965461	5.96E-09	4.86E-08	LCL	[21]
rs11732650	106973680	6.83E-09	5.02E-08	LCL	
rs11733225	106924812	2.34E-09	8.43E-08	LCL	
rs11733287	106924788	2.32E-09	8.47E-08	LCL	
rs11733654	106950159	0.0358	4.62E-08	LCL	
rs11735851	106916703	1.90E-09	8.95E-08	LCL	
rs11736859	106928234	2.86E-09	8.08E-08	LCL	
rs12374256	106836810	1.88E-09	1.04E-07	LCL	
rs12374256	106836810	1.88E-09	0.047 (posterior probability)	LCL	[40]
rs17036090	106813023	3.84E-08	1.70E-07	Cortex (Brain)	[42]
rs17036139	106852106	1.25E-09	0.057 (posterior probability)	LCL	[40]
rs17036139	106852106	1.25E-09	1.07E-07	Cortex (Brain)	[42]
rs17036142	106854185	1.11E-09	0.049 (posterior probability)	LCL	[40]
rs17036142	106854185	1.11E-09	2.56E-08	Cortex (Brain)	[42]
rs17036225	106929541	3.33E-09	4.88E-08	Cortex (Brain)	
rs6820671	106833270	1.31E-05	2.88E-05	Liver	[43]
rs7676975	106983260	6.75E-09	5.18E-08	LCL	[21]

### Correlation of GSTCD and *INTS12* mRNA levels in the lungs with percent predicted FEV_1_

**Figure 5:**
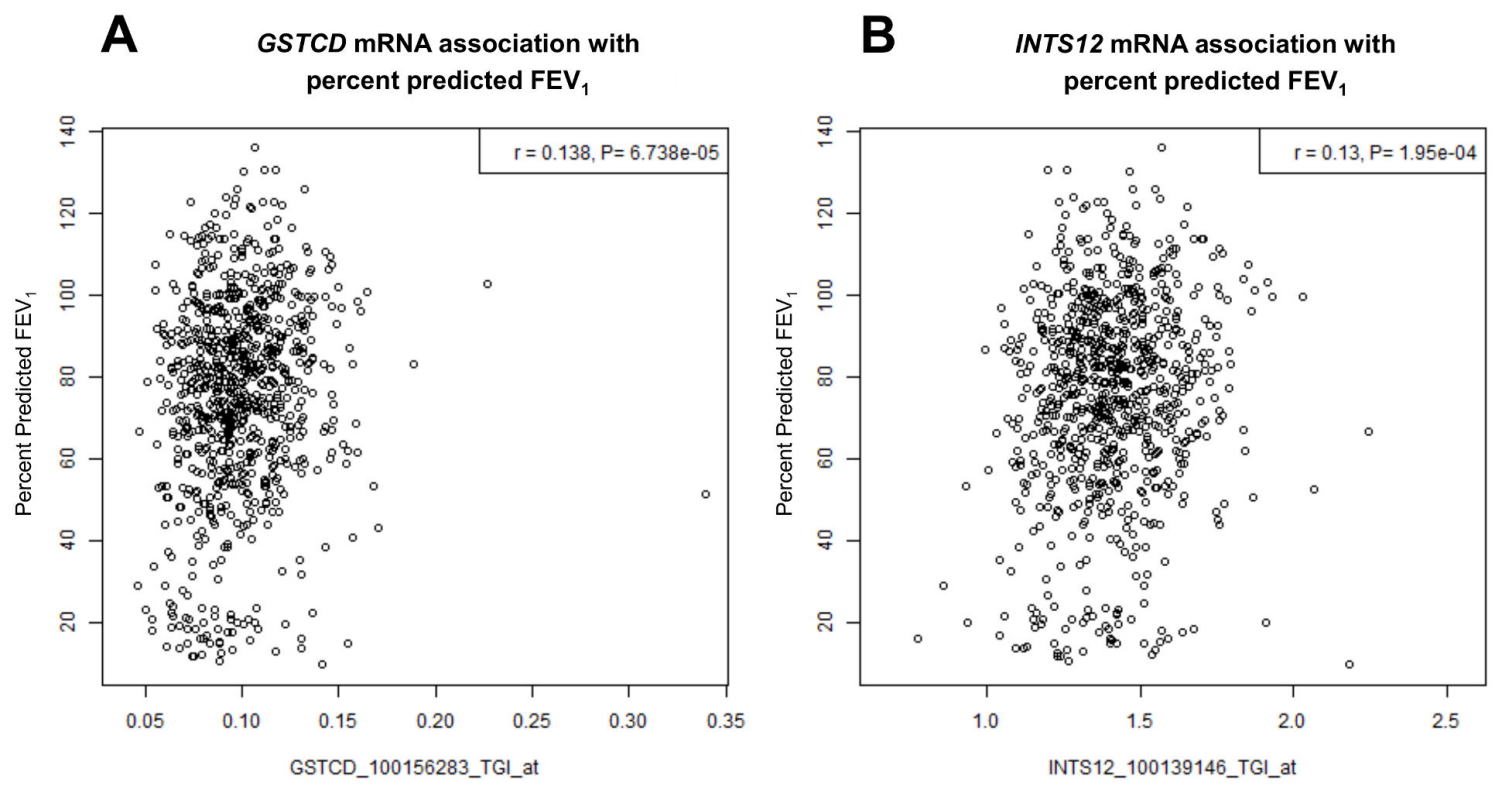
Correlation of *GSTCD* (A) and *INTS12* (B) lung mRNA levels with percent predicted FEV_1_ in the lungs of 848 individuals (see Table S5 for patient demographics).

Three probe sets targeting *GSTCD* and one probe set for *INTS12* were utilised in the lung eQTL study. The correlation measures of these probe sets with percent predicted FEV_1_ are shown in Figure 5. One of the *GSTCD* probe sets (100156283_TGI_at, r=0.14, *P*=6.74x10^-5^) and the *INTS12* probe set (r=0.13, *P*=1.95x10^-4^) show reasonable correlation with percent predicted FEV_1_, although the correlation was more statistically significant with the *GSTCD* probe set. Moreover, the direction of correlations is such that higher mRNA levels correlate with better lung function indicated by higher percent predicted FEV_1_. Table S5 shows the demographics of the individuals which participated in this study.

### Expression of GSTCD and INTS12 protein in human lung

Having dissected the genetic architecture of *GSTCD* and *INTS12* and explored their regulation at the mRNA level, we next investigated expression at the protein level in the human airways. In addition to utilising samples from non-diseased sections of human lungs, we assessed expression of both proteins in individuals with COPD and also in human lung at a range of fetal developmental stages.

**Figure 6:**
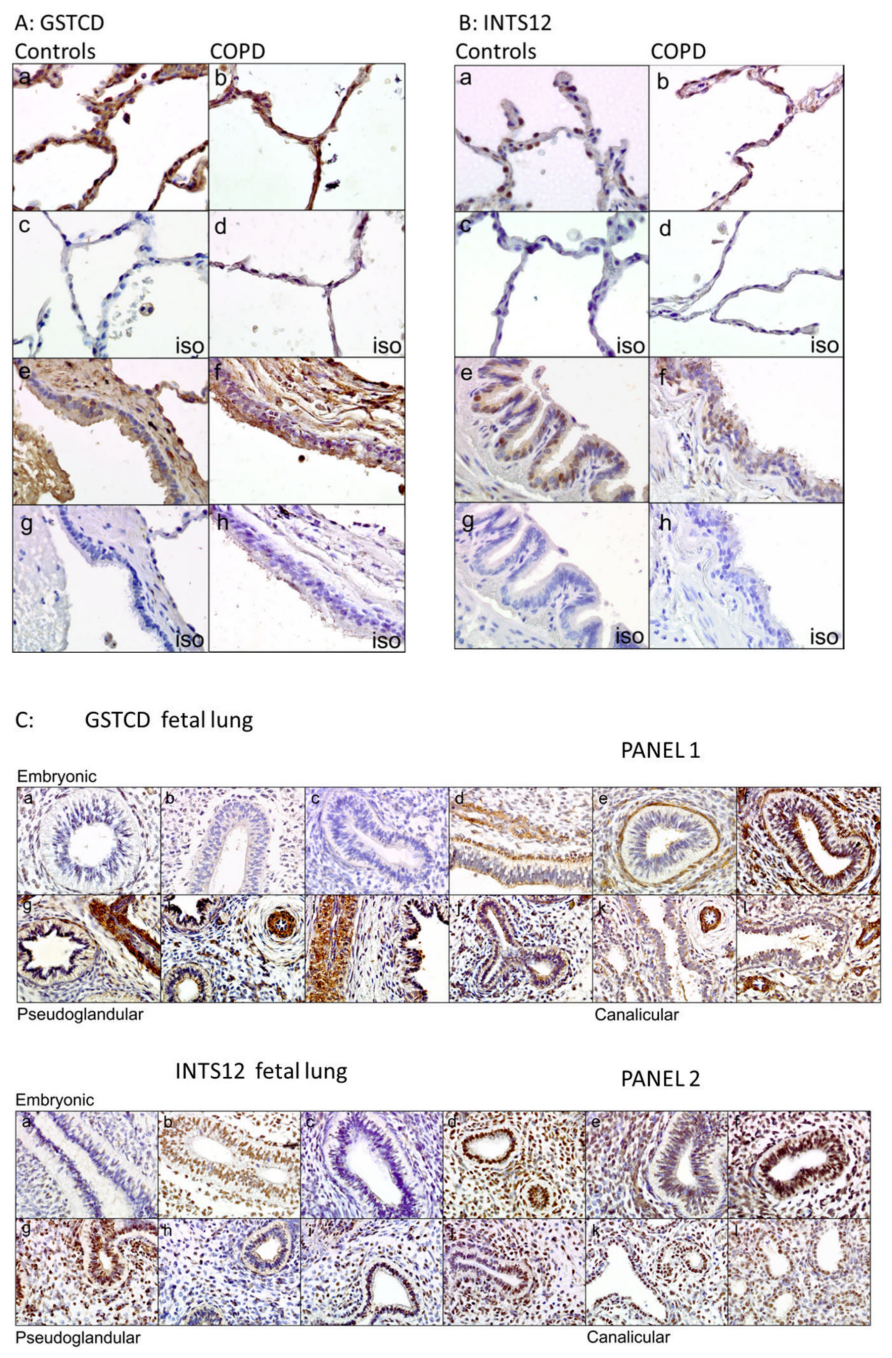
GSTCD and INTS12 protein expression in human tissue. Immunohistochemistry studies assessed GSTCD and INTS12 protein expression in tissue sections from controls, individuals with COPD and from a range of fetal developmental stages. All images x40 magnification. **A**: Representative images of GSTCD expression in lung tissue from three healthy donors (images a and e) with matched isotype controls (iso, images c and g) and in lung tissue from three donors with COPD (images b and f) with matched isotype controls (iso, images d and h). **B**: Representative images of INTS12 expression in lung tissue from three control donors (images a and e) with matched isotype controls (iso, images c and g) and in lung tissue from three donors with COPD (images b and f) with matched isotype controls (iso, images d and h). **C**: Panel 1: GSTCD expression in tissue from human fetuses at a range of developmental stages: embryonic (19 days, a and b; 21 days, c and d; 23 days, e and f); pseudoglandular (10 weeks, g and h; 12 weeks, i and j); canalicular (17 weeks, k; 19 weeks, l). Expression was observed to be increased through the pseudoglandular stage. Panel 2: INTS12 expression in the same panel of tissue samples as described above. Isotype controls were all negative (data not shown).

Figure 6 shows representative images of the expression of GSTCD (6A) and INTS12 (6B) in control donors (panels a and e) and in lung samples from individuals with COPD (panels b and f). In controls, GSTCD was observed to be expressed ubiquitously, being most evident in the pneumocytes of alveolar regions (Figure 6A, image a) and in the bronchial epithelium (Figure 6A, image e). In all cell types, expression was observed to be predominantly cytoplasmic. INTS12 expression in controls and individuals with COPD was most evident in the nuclei of alveolar pneumocytes (Figure 6B, images a and b) and also in bronchial epithelial cells (Figure 6B, images e and f).

When expression was investigated through different stages of fetal development, the earliest embryonic samples (19 days) exhibited weak nuclear and cytoplasmic immuno-positivity for GSTCD (Figure 6C, top panel, images a and b), while embryonic samples at 23 days development (images e and f) showed strong protein expression in the developing blood vessels and the outer region of the developing bronchioles. Strong GSTCD expression was observed throughout the pseudoglandular stage (images g-j), where expression was noted in the nuclei of the developing pneumocytes of the early bronchiole. Although high protein expression was still observed in the blood vessels in tissue from the canalicular stage, at this stage expression in the pneumocytes appeared to be reduced compared with earlier developmental stages (images k-l).

When comparisons were made between samples, INTS12 expression remained generally consistent throughout all 3 developmental stages of fetal lung development, although in 2 out of the 6 early embryonic samples expression was somewhat reduced (Figure 6C, panel 2, images a and c). Expression was localised to the nucleus in most airway cell types (Figure 6C, panel 2). Expression profiling of *GSTCD* and *INTS12* was also investigated using the BioGPS portal and this revealed ubiquitous expression [22].

### Comparison of GSTCD and INTS12 mRNA and protein levels through lung development

To assess whether the expression of GSTCD and INTS12 through lung development at the protein level correlated with that found at the mRNA level, a previously reported dataset was mined to specifically interrogate these genes [23]. This dataset contains samples analysed using gene expression arrays to investigate expression patterns in the developing human lung, specifically throughout the pseudoglandular (7-16 weeks) and the canalicular (17-26 weeks) stages. While *INTS12* expression was not observed to be altered significantly between these stages, interestingly *GSTCD* expression significantly decreased with age between the pseudoglandular and canalicular stages (Table S6) as we also had observed at the protein level (Figure 6C, panel 1, pseudoglandular images g-j, canalicular images k-l).

## Discussion

The aims of the studies described in this manuscript were to (i) define the genetic architecture surrounding the SNPs at the *GSTCD*/*INTS12* locus at 4q24 previously found to be strongly associated with lung function, (ii) assess how *GSTCD* and *INTS12* gene expression is regulated, (iii) analyse GSTCD and INTS12 protein expression in non-diseased lung tissue and lung tissue from individuals with COPD and (iv) assess whether GSTCD or INTS12 protein expression changes during fetal development.

The main conclusions were that lung function associated SNPs in the region act as eQTL for *INTS12* in multiple tissue types including lymphoblastoid cell lines (LCL), brain and liver and that both genes were widely expressed in cell types relevant to airway function, including airway smooth muscle cells and epithelial cells with evidence for coordinated gene expression of *GSTCD* and *INTS12*. Finally mRNA levels in the lung of both genes were correlated with lung function. It seems likely that the association signal in this region is explained by altered gene expression of *INTS12* or *GSTCD*, or potentially both genes.

Existing evidence suggests that transcript levels of *GSTCD* are associated with variability in lung function from a study that investigated lung mRNA expression levels among individuals with variable degrees of airway obstruction [15]. This was confirmed when we utilised data from a larger sample set that aimed to identify lung eQTL and where lung function measures were available on the majority of participants (n=848) (Figure 5). Findings from this study suggest a strong correlation between a probe set targeting *GSTCD* with percent predicted FEV_1_ and also, between the *INTS12* probe set and percent predicted FEV_1_.

It was particularly interesting that GSTCD protein expression was observed to be altered through lung development, decreasing between the pseudoglandular and canalicular stages. Importantly, this correlates with a decrease in *GSTCD* at the mRNA level between these same developmental stages, as revealed by mining and specifically analysing the results of a study previously reported by Melén et al. [23]. That protein and mRNA levels of GSTCD are relatively higher in the pseudoglandular stage when compared with the canalicular stage is significant as it is through this stage of development that airway smooth muscle cells, mast cells, T-lymphocytes and dendritic cells all begin to appear within the lung parenchyma [23]. Conversely, the reduction in GSTCD in the canalicular phase coincides with alteration of the epithelium and the surrounding mesenchyme.

Very little is known about the potential function of GSTCD. As suggested by the name, part of this protein shares homology with a GST motif which is present in a number of other proteins. A protein homology search using NCBI BLAST identified a number of other proteins sharing similar motifs with GSTCD variants (Table S1A and 1B) with the closest match being Eukaryotic Translation Elongation Factor 1, also known as Aminoacyl-tRNA Synthetase-Interacting Multifunctional Protein 3 (AIMP3). AIMP3 is an auxiliary component of the macromolecular aminoacyl-tRNA synthase complex which is reported to act as a molecular hub, linked to diverse signalling pathways [24]. Also found to share homology with GSTCD were isoforms of the Titin protein. Titin, previously known as Connectin, is important in striated muscle contraction although the expression and function of specific Titin isoforms have also been reported in a range of smooth muscle types [25,26]. Interestingly, we also found homology with human Chloride intracellular channel protein (hCLIC) 6. The role of hCLIC proteins in the airways is unknown, although chloride homeostasis has been shown to be important in a range of respiratory conditions such as Cystic Fibrosis.

INTS12 is the smallest subunit of the Integrator complex [14] that associates with the C-terminal domain of the largest subunit of RNA polymerase II (RNAPII) at the promoter of small nuclear (sn) RNA genes, where it mediates 3’ end processing of snRNAs. RNAi-mediated deletion of INTS12 in *Drosophila* S2 cells leads to snRNA mis-processing [27], suggesting a key role for INTS12 within the Integrator complex. INTS12 possesses a highly conserved PHD-type zinc finger domain (a motif often found in proteins involved in gene regulation), but surprisingly this feature is not necessary for the snRNA processing function of INTS12 in S2 cells [28]. Instead, a short 45 amino acid N-terminal domain appears both necessary and sufficient to re-establish Integrator function in INTS12-depleted cells and to mediate the binding and stabilisation of the larger INTS1 subunit [28]. A protein homology search did not provide additional insight into previously unrealised function (Table S1C). The role of INTS12 in the mammalian respiratory system has yet to be investigated, but a fundamental role for INTS12 in mammalian cellular function is suggested by preliminary evidence of pre-weaning lethality in transgenic mice lacking the *INTS12* gene (http://www.sanger.ac.uk/mouseportal/search?query=colonyprefix:MALX).

From both experimental and *in silico* searches, some interesting observations can be made regarding the arrangement and regulation of *GSTCD* and *INTS12*. The data presented suggest that under certain conditions in cells derived from adult lung tissue, *GSTCD* and *INTS12* are co-expressed. This was observed in the Q-PCR expression studies performed in airway-related cell types and PBMC (Figure 3B) and was confirmed in microarray data from the lung eQTL study (Figure 3C). In addition the expression changes in response to TGFβ1 treatment also seem to be coordinated (Figure 4). Data from Zebrafish suggest that the mean correlation coefficient *r* of all the neighbouring gene pairs is 0.075, compared to r=0.03 in a randomised genome of the same genes and expression values [29]. Thus our observed correlation coefficient of 0.64 suggests that *GSTCD* and *INTS12* are correlated to a greater extent than would be anticipated by chance for neighbouring genes.

The coordinated expression among GWAS signal is interesting. It may suggest that trait associated variants reside in regulatory regions that control the expression of multiple genes, and it is possible that these regulated genes are part of molecular pathways or networks underlying the trait. Furthermore, the co-expression could be due to genes sharing a bidirectional promoter, which are common in the human genome; it is estimated that divergent genes regulated by these account for 11% of all human genes [30,31]. Bidirectional promoters typically (i) lack TATA boxes (only 8% of bidirectional promoters contain a TATA box), (ii) have a high GC content (66%), (iii) are enriched in CpG islands (77%) [31,32], and (iv) about two thirds of non-overlapping bidirectional promoters were found to be shorter than 300 bp in length [31]. The *GSTCD/INTS12* region exhibits many of these characteristics: (i) they are divergent genes transcribed in opposite directions, (ii) they were shown to be expressed in all airway cell types tested, (iii) mRNA expression shows some degree of correlation, (iv) the regions upstream of their TSS lacked TATA boxes, and (v) the UCSC Genome Browser suggests the intergenic region contains a CpG island and has histone modification activity indicating active transcription. However, the lack of a clear correlation between GSTCD and INTS12 expression patterns during lung development *in utero* suggests additional, gene-specific regulatory pathways may also exist.

The observed regulation by TGFβ1 is of particular relevance within the context of several airway diseases, particularly COPD and Idiopathic Pulmonary Fibrosis, in which TGFβ1 is a key driver of fibrosis and extracellular matrix deposition. Perhaps surprisingly, *in silico* analyses of putative transcription factor binding sites did not reveal any matches between the intergenic *GSTCD*/*INTS12* shared promoter region and Smad-induced transcription sites. However, interestingly we did identify a number of other matches to transcription factors including Serum Response Factor (SRF), NFκB, Glucocorticoid Receptor (GR) and STAT3 which gives potential mechanistic insight into regulatory pathways for these genes (see Table S3). In human lung fibroblasts, SRF expression is dramatically increased following exposure to TGFβ [33] thus this is a likely mechanism for the TGFβ1-induced increase in *GSTCD* and *INTS12* mRNA in airway smooth muscle cells. STAT3 has recently been suggested to be a central mediator of pulmonary fibrosis *via* dysregulation of epithelial-mesenchymal communication [34]. GR-mediated signalling occurs *via* both gene repression and activation and it is unclear which contributes to the anti-inflammatory effects of glucocorticoids. However GR is able to associate directly with NFκB, for which a consensus site was also observed in the intergenic *GSTCD*/*INTS12* region.

Taken together, the evidence presented in this paper provides the first insight into the expression and regulation of *GSTCD* and *INTS12* in the airways. Whilst other genes at this locus (notably *NPNT*) may also potentially contribute, the evidence provided here suggests an important role for *GSTCD* and/or *INTS12*. Knowledge of the transcriptional and translational regulation and expression of these genes should provide important insights into the underlying cause of the observed strong association signal with lung function at this locus on 4q24.

## Materials and Methods

### Ethics statement

All work was conducted with full ethical approval from the sites involved. Details are given in the following sections.

### Cell culture, treatment and preparation of total RNA

Primary human airway smooth muscle (HASM) cells were isolated and prepared from three different donors then cultured as described previously [35]. Ethical approval for the use of primary cells was obtained from the Nottingham University Hospitals local ethical committee (ref. EC00/165). Written informed consent from the donor was obtained for use of this sample in research.

Undifferentiated, adherent human bronchial epithelial cells (HBEC) from three donors were commercially sourced from Lonza (Basel, Switzerland), and cultured in bronchial epithelial growth medium (BEGM) as per manufacturer’s instructions.

For analysis of altered gene expression *via* exposure to a range of stimuli, cells were grown to 70 80% confluency and the media changed to serum-free DMEM (for HASM) or BEGM lacking hydrocortisone, Epithelial Growth Factor and epinephrine (for HBEC) for 24h. Cells were exposed to the following stimuli for 4h or 24h at the concentrations given: TGFβ1 (10ng/ml), forskolin (10µM), U0126 (10µM), bradykinin (1µM) and Lipopolysaccharide (LPS) (*E. coli* 026:B6) (1µg/ml). All stimuli were obtained from Sigma Aldrich (Gillingham, UK).

For downstream analysis, cells were lysed and RNA extracted using silica columns (RNeasy mini kit, Qiagen, Crawley, UK). Total RNA was commercially obtained for peripheral blood mononuclear cells (PBMC) of three donors from 3H Biomedical (Uppsala, Sweden), and lung RNA from three donors was obtained from Applied Biosystems/Ambion (Warrington, UK).

### 5’ and 3’ Rapid Amplification of cDNA Ends (RACE)

RACE-ready cDNA was synthesised from total RNA (1µg) from lung, HASM, undifferentiated HBEC and PBMC using the GeneRacer kit as directed (Invitrogen, Paisley, UK). 5' and 3’ RACE used GeneRacer primers and gene-specific nested primers for amplification. PCR products generated were also cloned to further clarify the exon structure of the gene. Both RACE and PCR products were sequenced using BigDye 3.1 (Applied Biosystems) in conjunction with an ABI 310 DNA sequencer. Sequences were aligned to the human database using the BLAST alignment tool (blast.ncbi.nlm.nih.gov).

### Real-time PCR (Q-PCR)

mRNA levels of *GSTCD*/*INTS12* were quantified using a series of Q-PCR assays. Amplicons spanning exons 5–6 of *GSTCD* and exons 7–8 of *INTS12* were used with FAM/TAMRA labelled TaqMan probes. cDNA was synthesised using Superscript II (Invitrogen) and random hexamer primers as per manufacturer’s instructions. RNA (1µg) was used in a 20µl reverse transcription reaction Q-PCR was performed using TaqMan gene expression master mix (Applied Biosystems) and 18S ribosomal RNA endogenous control (Applied Biosystems). Data were corrected to the 18S housekeeper using the 2^-ΔCt^ method and normalised to lung mRNA expression using the 2^-ΔΔCt^ method.

### Immunohistochemistry

Three non-diseased adult lung samples and three lung samples from individuals with clinically diagnosed COPD were collected from the Nottingham Health Science Biobank (Nottingham, UK) with the required ethical approval (08/H0407/1). Twelve fetal tissue samples were obtained from The Human Developmental Biology Resource (Newcastle upon Tyne and London, UK, www.hdbr.org) at diverse stages of development, specifically 19, 21 and 23 days and 10, 12, 17 and 19 weeks post-conception. The Human Developmental Biology Resource is a tissue bank which obtained ethical approval from the NRES Committee London – Fulham (08/H0712/34). Samples were consented for with written informed consent from the donor or next of kin, in accordance with national banking procedures and the UK Human Tissue Act (2004). For all samples, 4µm whole tissue sections on glass slides were de-paraffinized in Histo-clear (National Diagnostics, Dublin, Ireland) and hydrated using decreasing concentrations of ethanol. Antigen retrieval was performed in a steamer for 20 minutes in sodium citrate buffer (pH 6.0), followed by an endogenous peroxidase block for 5 minutes (Dako, Cambs, UK). Slides were incubated with either a mouse polyclonal anti-GSTCD antibody (H00079807, Abnova, Taiwan, China) (1:100) or a rabbit polyclonal anti-INTS12 antibody (HPA035772, Sigma Prestige Antibodies, Dorset, UK) (1:800) for 1 hour at room temperature. Additional slides were stained in parallel with normal mouse or rabbit IgG as respective matched isotype controls (Invitrogen). The Dako Chemate Envision Detection Kit (Dako) with DAB chromogen was used for detection. Sections were then counterstained with Mayer’s Haematoxylin (Surgipath, Cambs, UK), dehydrated and a coverslip mounted using Vectamount (Vector Laboratories, Peterborough, UK). Human liver or tonsil tissues were used as positive controls for GSTCD and INTS12 staining, respectively, whilst a negative control consisted of primary antibody substituted for antibody diluent. Results were visualised using an Olympus BX14 light microscope.

### Statistical analysis

Differences in expression in Q-PCR experiments were evaluated using ANOVA and Dunnett’s or Tukey’s post hoc test as appropriate. Data were analysed using Prism v.5.01 (GraphPad software, La Jolla, CA). *P*<0.05 was considered a statistically significant threshold.

### Bioinformatics

Protein BLAST, BLASTP (http://blast.ncbi.nlm.nih.gov/Blast.cgi?PAGE=Proteins), was used to align homologous protein sequences against GSTCD and INTS12 protein isoforms obtained from NCBI.

The region containing *GSTCD*-*INTS12* was also investigated using the UCSC genome Browser [36] (accessed at http://genome.ucsc.edu/ in January 2013). More specifically, annotations features from the ENCODE (The Encyclopaedia of DNA Elements) Browser tracks [17] were used based on experimental data from the ENCODE Project Consortium [37].

Publically available data [23,38] were utilised to see whether *GSTCD* or *INTS12* was differentially expressed during normal human lung development. Previously, human fetal lung tissues were obtained from National Institute of Child Health and Human Development tissue databases and microarray profiled to investigate the expression spanning different gestational ages. RNA samples from 38 subjects (estimated gestational age 7-22 weeks or 53-154 days post-conception) i.e. pseudoglandular (gestational age, 7-16 weeks) and canalicular (17-26 weeks) stages of development were included within the dataset. These data are available at NCBI Gene Expression Omnibus (GEO, http://www.ncbi.nlm.nih.gov/geo), GSE14334. The dataset was mined using Affymetrix probe sets; 220063_at, 1554518_at, 241126_at and 235387_at for *GSTCD* and 218616_at for *INTS12* expression.

The presence of eQTL at 4q24 for *GSTCD* and *INTS12* was investigated using the publically available eQTL datasets. The lymphoblastoid cell lines eQTL website at http://www.hsph.harvard.edu/liming-liang/software/eqtl/ was used [21]. In addition, eQTL were also investigated using the Pritchard Lab eQTL website from the University of Chicago at http://eqtl.uchicago.edu/Home.html which hosts a browser summarising results from a collection of published eQTL studies.

### Lung eQTL consortium methodologies

#### Subjects and genomic dataset

Non-tumour lung tissues were collected from patients who underwent lung resection surgery at three participating sites: Laval University (Quebec City, Canada), University of Groningen (Groningen, The Netherlands), and University of British Columbia (UBC, Vancouver, Canada). Whole-genome gene expression and genotyping data were obtained from these specimens. Gene expression profiling was performed using an Affymetrix custom array testing 51,627 non-control probe sets and normalised using RMA [39]. Genotyping was performed using the Illumina Human1M-Duo BeadChip array. Following standard microarray and genotyping quality controls, 1,111 patients were available including 409 from Laval, 363 from Groningen, and 339 from UBC. At Laval, lung specimens were collected from patients undergoing lung cancer surgery and stored at the “Institut Universitaire de Cardiologie et de Pneumologie de Québec” (IUCPQ) site of the Respiratory Health Network, Tissue Bank of the “Fonds de recherche du Québec – Santé” (www.tissuebank.ca). Written informed consent was obtained from all subjects and the study was approved by the IUCPQ ethics committee. At Groningen, lung specimens were provided by the local tissue bank of the Department of Pathology and the study protocol was consistent with the Research Code of the University Medical Center Groningen and Dutch national ethical and professional guidelines (“Code of conduct; Dutch Federation of Biomedical Scientific Societies”; http://www.federa.org). At Vancouver, the lung specimens were provided by the James Hogg Research Center Biobank at St Paul’s Hospital and subjects provided written informed consent. The study was approved by the ethics committees at the UBC-Providence Health Care Research Institute Ethics Board.

#### Correlation of mRNA expression levels of *GSTCD* and *INTS12*

*GSTCD* was evaluated by three probe sets on the Affymetrix array, while a single probe set was tested for *INTS12*. The 1,111 individuals that passed all quality controls filters were included in the analyses. The relationship between the mRNA expression values of the different probe sets testing *GSTCD* and *INTS12* was assessed using Pearson’s correlations.

#### Lung eQTL analysis for *GSTCD* and *INTS12*

eQTL analyses were performed in the 1,111 subjects. SNPs located 50 kilobases up- and down-stream of *GSTCD* and *INTS12* were evaluated for association with their respective probe sets. Age, gender, smoking status, and the study site were included as covariates. Significant eQTL were those passing Bonferroni correction considering the number of SNPs tested for each gene.

#### Correlation of transcript levels with lung function

Correlations of transcript levels of *GSTCD* and *INTS12* in the lung with percent predicted FEV_1_ were investigated using Pearson’s correlation. These analyses were performed with 848 subjects (a sub-set of the 1,111 available), for whom lung function data were available and which excluded individuals with airway disease which was non-obstructive or not due to smoking. The demographics of these 848 individuals are included in Table S5.

## Supporting Information

Figure S1 Annotated first exons of GSTCD Variant 2, Variant 3 and *INTS12* Variant 2 showing transcription start sites (TSSs) identified by 5` RACE. Nucleotide sequence of the first exon of GSTCD Variant 2 (A), Variant 3 (B) and *INTS12* Variant 2 (C) showing in all cases the NCBI RefSeq (build 37) start site and the start of intron 1 (accessed March 2013). Highlighted in green are the TSSs identified by 5’ RACE with the red number above indicating the number of clones observed to contain that specific TSS.
(DOCX)

Figure S2 RT-PCR attempting to amplify *GSTCD* Variant 1 (NM_001031720.2) using primers mapping to exons 1 and 3. No amplicon was detected after 40 cycles of PCR. Cycling conditions were as follows: 94°C for 3 minutes, 35 cycles of 94°C for 45 seconds, 55°C for 30 seconds, and 72°C for 90 seconds. GAPDH was used as a housekeeping gene and a product of the expected size of 508 bp is shown indicating the RT-PCR assay was successful.
(DOCX)

Figure S3 eQTL in the region containing *GSTCD* and *INTS12* at 4q24. (A) shows eQTL identified in lymphoblastoid cell lines using the eQTL website at http://www.hsph.harvard.edu/liming-liang/software/eqtl/ (Liang et al., Genome research **23**(4): 716-726) and (B) shows the eQTL identified using the Pritchard Lab eQTL website at http://eqtl.uchicago.edu/cgi-bin/gbrowse/eqtl/. Both (A) and (B) show that eQTL exist for *INTS12* in the region.
(PPTX)

Figure S4 Lung eQTL for *GSTCD* and *INTS12*. Shown are the genotyped and imputed Single Nucleotide Polymorphism (SNP) associations with *GSTCD* (purple) and *INTS12* (green) probe sets expression levels in the lungs of 1,111 individuals. The scale on the left side Y axis shows the eQTL -log_10_ *P* values, and the scale on the right hand side Y axis shows the FEV_1_ association -log_10_ *P* values. The red X represents the sentinel SNP rs10516526 for association with FEV_1_. The three probe sets for *GSTCD* were merged into gene level eQTL.
(DOCX)

Table S1 **A**. **Results from a Protein Homology Search of GSTCD variant 1 (Accession NP_001026890.2)**. Search was performed 30/01/2013 using BLAST (Basic Local Alignment Search Tool) (http://blast.ncbi.nlm.nih.gov/Blast.cgi). Accession: protein accession number; Chr: Chromosome; E value: Expect value (E), a parameter that describes the number of hits one can "expect" to see by chance when searching a database of a particular size. **B**. **Results from a Protein Homology Search of GSTCD variant 2 (Accession NP_079027.2)**. Search was performed 30/01/2013 using BLAST (Basic Local Alignment Search Tool) (http://blast.ncbi.nlm.nih.gov/Blast.cgi). Accession: protein accession number; Chr: Chromosome; E value: Expect value (E), a parameter that describes the number of hits one can "expect" to see by chance when searching a database of a particular size. **C**. **Results from a Protein Homology Search of INTS12 (Accession NP_001135943.1)**. Search was performed 30/01/2013 using BLAST (Basic Local Alignment Search Tool) (http://blast.ncbi.nlm.nih.gov/Blast.cgi). Accession: protein accession number; Chr: Chromosome; E value: Expect value (E), a parameter that describes the number of hits one can "expect" to see by chance when searching a database of a particular size.
(DOCX)

Table S2 **A**. **100 “resistant” smokers whole exome re-sequencing**. An exome re-sequencing project (results unpublished) identified 1 non-synonymous SNP and 1 synonymous SNP in the coding regions of *INTS12* which were listed in dbSNP v137 but which were not in the 1000 Genomes Project imputation reference panel. Both SNPs occurred as a singleton call (1 heterozygote, no ALT allele homozygotes) in the 100 individuals. rs146172950 is listed as a triallelic SNP in dbSNP137 with reference allele C and alternative allele T (frequency 0.049%) originally identified and submitted by the NHLBI Exome Sequencing Project and alternative allele A (frequency 0.016%) subsequently identified and submitted by the 1000 Genomes Project. The alternative allele T was observed in the 100 resistant smokers exome sequencing project. rs144650579 is listed in dbSNP137 with reference allele T and alternative allele C (frequency 0.085%) originally identified and submitted by the NHLBI Exome Sequencing Project. **B**. **Targeted sequencing of *GSTCD*/*INTS12* region**. A targeted sequencing study (results unpublished) identified two intronic SNPs in *GSTCD* that do not appear in 1000 Genomes Project. All the SNPs identified in *INTS12* appear in 1000 Genomes Project imputation reference panel. rs113987854 is listed in dbSNP137 with reference allele T and alternative allele A originally identified and submitted by Complete Genomics. No population frequency is available for this SNP (only counts based on one individual have been submitted to dbSNP137).
(DOCX)

Table S3 Transcription Factor sites identified via the ENCODE project Chip-Seq assay in the region: chr4:106609150-106654787 (hg build37). ChromStart: Start position in chromosome; ChromEnd: End position in chromosome; Name: name of transcription factor; Score: Score from 0-1000 (score for each peak, which reflects the posterior probability of the peak belonging to the irreproducible group). Search performed 30/01/2013.
(DOCX)

Table S4 ENCODE functional annotations of INTS12 eQTL using HaploReg V2. Shown are the predicted functional annotation of the SNPs in terms of spanning a promoter, enhancer site, DNase hypersensitivity site, proteins bound, or the motifs changed and the corresponding cell type in which this was detected. Gene name indicates the name of the gene spanned by the SNP.
(XLSX)

Table S5 Clinical characteristics of 848 individuals participating in the eQTL study. Pre: pre-bronchodilator; Post: post-bronchodilator; Laval (Laval University, Canada), Groningen (University of Groningen, The Netherlands) and UBC (University of British Columbia, Canada) refer to the cohorts recruited from each centre. Data are presented as mean ± standard deviation.
(DOCX)

Table S6 Fetal lung gene array data comparing *GSTCD* and *INTS12* expression during pseudoglandular and canalicular stages of lung development. logFC: log-fold difference between 2 experimental groups; Average expression is between all samples; t: t-statistic describing differential expression; *P* Value: Unadjusted *P* value; Adjusted p value controls for false discovery rate; Beta-coefficient: log-odds ratio. n/s: no significance observed. Significant effects are shown in bold and italicised.
(DOCX)
