# Supplementary material for: GSTCD and INTS12 Regulation and Expression in the Human Lung
Source: PLoS One. 2013 Sep 18;8(9):e74630. doi: 10.1371/journal.pone.0074630 (PMC3776747; doi:10.1371/journal.pone.0074630)
Supplement: Figure S1 — Nucleotide sequence of the first exon of GSTCD Variant 2 (A), Variant 3 (B) and INTS12 Variant 2 (C) showing in all cases the NCBI RefSeq (build 37) start site and the start of intron 1 (accessed March 2013). Highlighted in green are the TSSs identified by 5’ RACE with the red number above indicating the number of clones observed to contain that specific TSS. (DOCX) [file pone.0074630.s001.docx]

**A**

**B**

**C**

Figure S1. Annotated first exons of *GSTCD* Variant 2, Variant 3 and *INTS12* Variant 2 showing transcription start sites (TSSs) identified by 5` RACE. Nucleotide sequence of the first exon of *GSTCD* Variant 2 (A), Variant 3 (B) and *INTS12* Variant 2 (C) showing in all cases the NCBI RefSeq (build 37) start site and the start of intron 1 (accessed March 2013). Highlighted in green are the TSSs identified by 5’ RACE with the red number above indicating the number of clones observed to contain that specific TSS.
