# Supplementary material for: GSTCD and INTS12 Regulation and Expression in the Human Lung
Source: PLoS One. 2013 Sep 18;8(9):e74630. doi: 10.1371/journal.pone.0074630 (PMC3776747; doi:10.1371/journal.pone.0074630)
Supplement: Figure S2 — No amplicon was detected after 40 cycles of PCR. Cycling conditions were as follows: 94°C for 3 minutes, 35 cycles of 94°C for 45 seconds, 55°C for 30 seconds, and 72°C for 90 seconds. GAPDH was used as a housekeeping gene and a product of the expected size of 508 bp is shown indicating the RT-PCR assay was successful. (DOCX) [file pone.0074630.s002.docx]

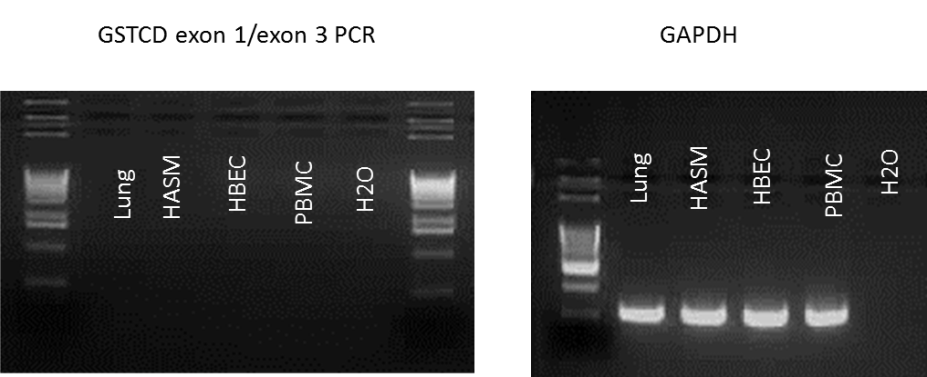


Figure S2. RT-PCR attempting to amplify *GSTCD* Variant 1 (NM_001031720.2) using primers mapping to exons 1 and 3. No amplicon was detected after 40 cycles of PCR. Cycling conditions were as follows: 94°C for 3 minutes, 35 cycles of 94°C for 45 seconds, 55°C for 30 seconds, and 72°C for 90 seconds. GAPDH was used as a housekeeping gene and a product of the expected size of 508 bp is shown indicating the RT-PCR assay was successful.
