# Supplementary material for: GSTCD and INTS12 Regulation and Expression in the Human Lung
Source: PLoS One. 2013 Sep 18;8(9):e74630. doi: 10.1371/journal.pone.0074630 (PMC3776747; doi:10.1371/journal.pone.0074630)
Supplement: Figure S3 — (A) shows eQTL identified in lymphoblastoid cell lines using the eQTL website at http://www.hsph.harvard.edu/liming-liang/software/eqtl/ (Liang et al., Genome research 23(4): 716-726) and (B) shows the eQTL identified using the Pritchard Lab eQTL website at http://eqtl.uchicago.edu/cgi-bin/gbrowse/eqtl/. Both (A) and (B) show that eQTL exist for INTS12 in the region. (PPTX) [file pone.0074630.s003.pptx]

## Slide 1
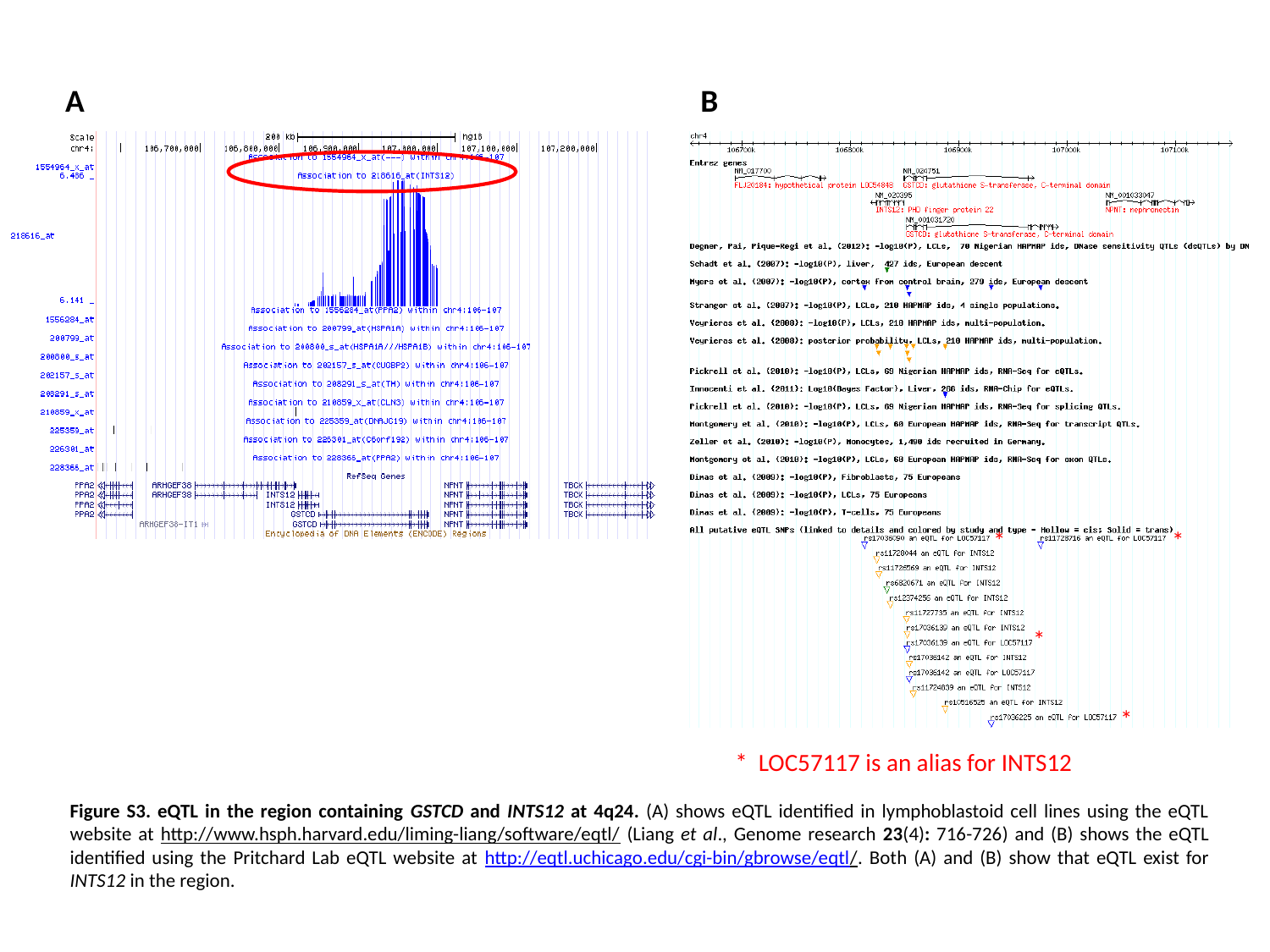

A
B
*
*
*
*
* LOC57117 is an alias for INTS12
Figure S3. eQTL in the region containing GSTCD and INTS12 at 4q24. (A) shows eQTL identified in lymphoblastoid cell lines using the eQTL website at http://www.hsph.harvard.edu/liming-liang/software/eqtl/ (Liang et al., Genome research 23(4): 716-726) and (B) shows the eQTL identified using the Pritchard Lab eQTL website at http://eqtl.uchicago.edu/cgi-bin/gbrowse/eqtl/. Both (A) and (B) show that eQTL exist for INTS12 in the region.
