# Supplementary material for: GSTCD and INTS12 Regulation and Expression in the Human Lung
Source: PLoS One. 2013 Sep 18;8(9):e74630. doi: 10.1371/journal.pone.0074630 (PMC3776747; doi:10.1371/journal.pone.0074630)
Supplement: Figure S4 — Shown are the genotyped and imputed Single Nucleotide Polymorphism (SNP) associations with GSTCD (purple) and INTS12 (green) probe sets expression levels in the lungs of 1,111 individuals. The scale on the left side Y axis shows the eQTL -log10 P values, and the scale on the right hand side Y axis shows the FEV1 association -log10 P values. The red X represents the sentinel SNP rs10516526 for association with FEV1. The three probe sets for GSTCD were merged into gene level eQTL. (DOCX) [file pone.0074630.s004.docx]

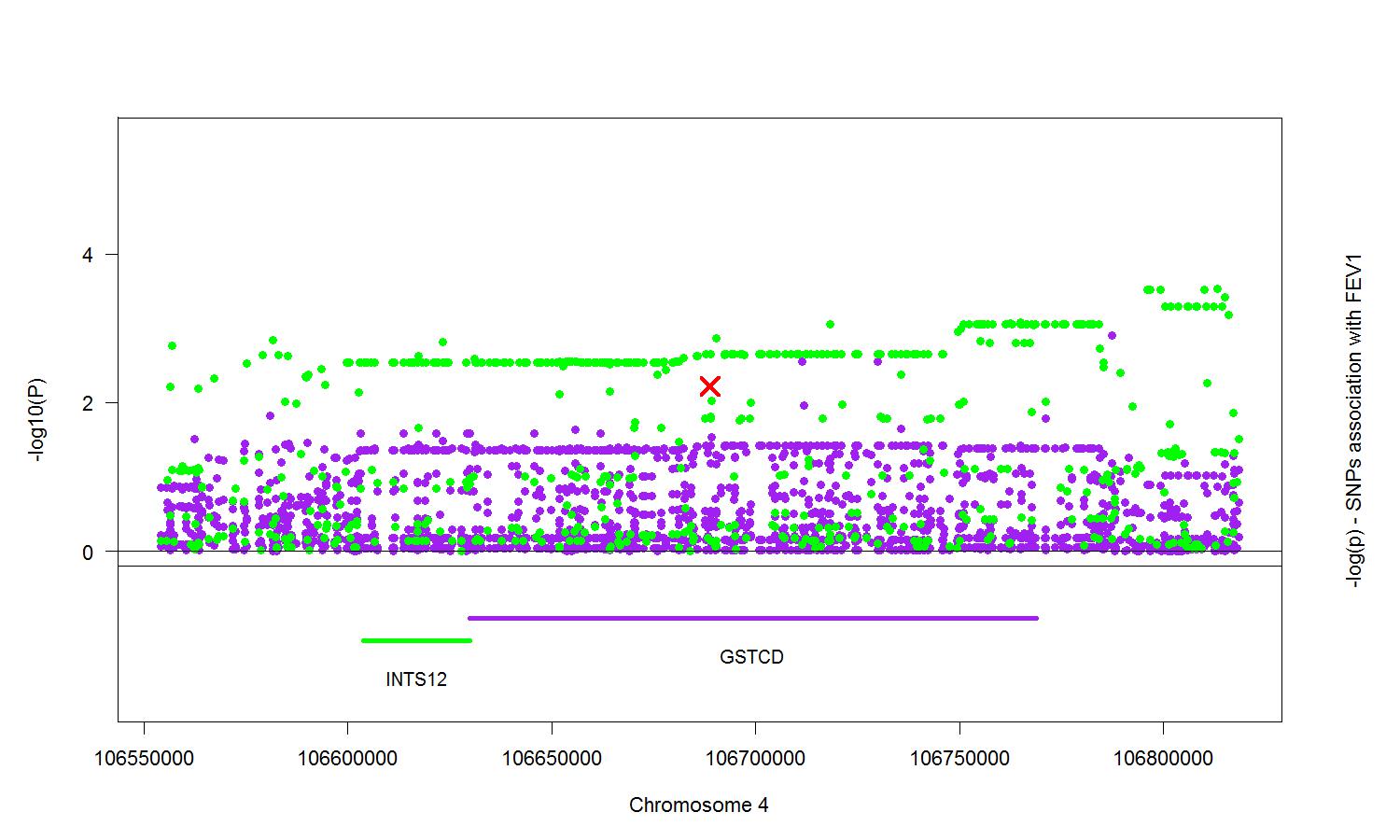


**Figure S4.** **Lung eQTL for *GSTCD* and *INTS12*.** Shown are the genotyped and imputed Single Nucleotide Polymorphism (SNP) associations with *GSTCD* (purple) and *INTS12* (green) probe sets expression levels in the lungs of 1,111 individuals. The scale on the left side Y axis shows the eQTL –log10 *P* values, and the scale on the right hand side Y axis shows the FEV_1_ association –log10 *P* values. The red X represents the sentinel SNP rs10516526 for association with FEV_1_. The three probe sets for *GSTCD* were merged into gene level eQTL.
