# Supplementary material for: GSTCD and INTS12 Regulation and Expression in the Human Lung
Source: PLoS One. 2013 Sep 18;8(9):e74630. doi: 10.1371/journal.pone.0074630 (PMC3776747; doi:10.1371/journal.pone.0074630)
Supplement: Table S1 — A. Results from a Protein Homology Search of GSTCD variant 1 (Accession NP_001026890.2). Search was performed 30/01/2013 using BLAST (Basic Local Alignment Search Tool) (http://blast.ncbi.nlm.nih.gov/Blast.cgi). Accession: protein accession number; Chr: Chromosome; E value: Expect value (E), a parameter that describes the number of hits one can "expect" to see by chance when searching a database of a particular size. B. Results from a Protein Homology Search of GSTCD variant 2 (Accession NP_079027.2). Search was performed 30/01/2013 using BLAST (Basic Local Alignment Search Tool) (http://blast.ncbi.nlm.nih.gov/Blast.cgi). Accession: protein accession number; Chr: Chromosome; E value: Expect value (E), a parameter that describes the number of hits one can "expect" to see by chance when searching a database of a particular size. C. Results from a Protein Homology Search of INTS12 (Accession NP_001135943.1). Search was performed 30/01/2013 using BLAST (Basic Local Alignment Search Tool) (http://blast.ncbi.nlm.nih.gov/Blast.cgi). Accession: protein accession number; Chr: Chromosome; E value: Expect value (E), a parameter that describes the number of hits one can "expect" to see by chance when searching a database of a particular size. (DOCX) [file pone.0074630.s005.docx]

**Table S1A. Results from a Protein Homology Search of GSTCD variant 1 (Accession NP_001026890.2).** Search was performed 30/01/2013 using BLAST (Basic Local Alignment Search Tool) (<http://blast.ncbi.nlm.nih.gov/Blast.cgi>). Accession: protein accession number; Chr: Chromosome; E value: Expect value (E), a parameter that describes the number of hits one can "expect" to see by chance when searching a database of a particular size.

| **Accession** | **Gene** | **Chr** | **Description** | **Score** | **E value** |
| --- | --- | --- | --- | --- | --- |
| **NP_004271** | EEF1E1 | 6 | Eukaryotic translation elongation factor 1 epsilon-1 V1 | 39.3 | 0.004 |
| **NP_003119.2** | SPTBN1 | 2 | Spectrin beta chain, non-erythrocytic isoform 1 | 33.5 | 0.76 |
| **NP_001254479.1** | TTN | 2 | Titin isoform IC | 33.1 | 1.1 |
| **NP_001243779.1** | TTN | 2 | Titin isoform N2BA | 33.1 | 1.1 |
| **NP_596869.4** | TTN | 2 | Titin isoform N2-A | 33.1 | 1.2 |
| **NP_597681.3** | TTN | 2 | Titin isoform novex-2 | 32.7 | 1.3 |
| **NP_003310.4** | TTN | 2 | Titin isoform N2-B | 32.7 | 1.4 |
| **NP_597676.3** | TTN | 2 | Titin isoform novex-1 | 32.7 | 1.4 |
| **NP_003768.2** | DNAH11 | 7 | Dynein heavy chain 11, axonemal | 32.0 | 2.1 |
| **NP_003655.3** | AP3B1 | 5 | AP-3 complex subunit beta-1 isoform 1 | 30.4 | 6.1 |
| **NP_149113.3** | CABS1 | 4 | Calcium-binding and spermatid-specific protein 1 | 30.0 | 6.4 |
| **NP_001258698.1** | AP3B1 | 5 | AP-3 complex subunit beta-1 isoform 2 | 30.4 | 6.7 |
| **XP_003960890.1** | LOC100361383 | X | PREDICTED: Extracellular matrix protein 2-like | 30.4 | 6.8 |
| **NP_006512.2** | TFE3 | X | Transcription factor E3 | 30.0 | 7.2 |
| **XP_003960077.1** | LOC100361383 | X | PREDICTED: Extracellular matrix protein 2-like | 30.0 | 7.3 |
| **XP_003403925.1** | LOC100509091 | X | PREDICTED: Extracellular matrix protein 2-like | 29.6 | 9.7 |

**Table S1B. Results from a Protein Homology Search of GSTCD variant 2 (Accession NP_079027.2).** Search was performed 30/01/2013 using BLAST (Basic Local Alignment Search Tool) (<http://blast.ncbi.nlm.nih.gov/Blast.cgi>). Accession: protein accession number; Chr: Chromosome; E value: Expect value (E), a parameter that describes the number of hits one can "expect" to see by chance when searching a database of a particular size.

| **Accession** | **Gene** | **Chr** | **Description** | **Score** | **E value** |
| --- | --- | --- | --- | --- | --- |
| **NP_004271** | EEF1E1 | 6 | Eukaryotic translation elongation factor 1 epsilon-1 Variant 1 | 39.3 | 0.003 |
| **NP_001243779.1** | TTN | 2 | Titin isoform N2BA | 33.1 | 0.87 |
| **NP_001254479.1** | TTN | 2 | Titin isoform IC | 33.1 | 0.96 |
| **NP_596869.4** | TTN | 2 | Titin isoform N2-A | 32.7 | 0.99 |
| **NP_597676.3** | TTN | 2 | Titin isoform novex-1 | 32.7 | 1.1 |
| **NP_003310.4** | TTN | 2 | Titin isoform N2-B | 32.7 | 1.1 |
| **NP_597681.3** | TTN | 2 | Titin isoform novex-2 | 32.7 | 1.1 |
| **NP_003768.2** | DNAH11 | 7 | Dynein heavy chain 11, axonemal | 32.3 | 1.5 |
| **NP_006512.2** | TFE3 | X | Transcription factor E3 | 30.0 | 5.9 |
| **NP_149113.3** | CABS1 | 4 | Calcium-binding and spermatid-specific protein 1 | 30.0 | 5.9 |
| **XP_003960077.1** | LOC100361383 | X | PREDICTED: Extracellular matrix protein 2-like | 30.0 | 6.4 |
| **XP_003960890.1** | LOC100361383 | X | PREDICTED: Extracellular matrix protein 2-like | 30.0 | 6.4 |
| **XP_003403925.1** | LOC100509091 | X | PREDICTED: Extracellular matrix protein 2-like | 29.6 | 9.3 |
| **NP_444507.1** | CLIC6 | 21 | Chloride intracellular channel protein 6 | 29.6 | 9.5 |

**Table S1C. Results from a Protein Homology Search of INTS12 (Accession NP_001135943.1).** Search was performed 30/01/2013 using BLAST (Basic Local Alignment Search Tool) (<http://blast.ncbi.nlm.nih.gov/Blast.cgi>). Accession: protein accession number; Chr: Chromosome; E value: Expect value (E), a parameter that describes the number of hits one can "expect" to see by chance when searching a database of a particular size.

| **Accession** | **Gene** | **Chr** | **Description** | **Score** | **E value** |
| --- | --- | --- | --- | --- | --- |
| **NP_077084.1** | PHF1 | 6 | PHD finger protein 1 isoform b | 50.8 | 2.00E-06 |
| **NP_002627.1** | PHF1 | 6 | PHD finger protein 1 isoform a | 50.4 | 2.00E-06 |
| **NP_001095272.1** | PHF21A | 11 | PHD finger protein 21A isoform a | 45.4 | 1.00E-04 |
| **NP_057705.3** | PHF21A | 11 | PHD finger protein 21A isoform b | 44.7 | 2.00E-04 |
| **NP_004500.3** | SP110 | 2 | sp110 nuclear body protein isoform a | 42.7 | 5.00E-04 |
| **NP_001184033.1** | MLL | 11 | Myeloid/lymphoid or mixed-lineage | 43.1 | 6.00E-04 |
| **NP_005924.2** | MLL | 11 | Myeloid/lymphoid or mixed-lineage | 43.1 | 6.00E-04 |
| **NP_536349.2** | SP110 | 2 | sp110 nuclear body protein isoform c | 42.7 | 6.00E-04 |
| **NP_031384.1** | MTF2 | 1 | Metal-response element-binding transcription factor 2 isoform a | 41.6 | 0.001 |
| **NP_001157863.1** | MTF2 | 1 | Metal-response element-binding transcription factor 2 isoform c | 40.8 | 0.002 |
| **NP_001157864.1** | MTF2 | 1 | Metal-response element-binding transcription factor 2 isoform b | 40.8 | 0.002 |
| **NP_038478.2** | BAZ2B | 2 | Bromodomain adjacent to zinc finger domain protein 2B | 38.1 | 0.017 |
| **NP_001009936.1** | PHF19 | 9 | PHD finger protein 19 isoform b | 36.2 | 0.035 |
| **NP_056989.2** | TRIM24 | 7 | Transcription intermediary factor 1-alpha isoform a | 36.6 | 0.053 |
| **NP_009168.4** | SP140 | 2 | Nuclear body protein SP140 isoform 1 | 36.6 | 0.054 |
| **NP_003843.3** | TRIM24 | 7 | Transcription intermediary factor 1-alpha isoform b | 36.6 | 0.059 |
| **NP_056466.1** | PHF19 | 9 | PHD finger protein 19 isoform a | 36.2 | 0.068 |
| **NP_005753.1** | TRIM28 | 19 | Transcription intermediary factor 1-beta | 35.0 | 0.14 |
| **NP_733751.2** | MLL3 | 7 | Histone-lysine-N-methyltransferase MLL3 | 35.0 | 0.16 |
| **NP_612411.4** | SP140L | 2 | Nuclear body protein SP140-like protein | 34.3 | 0.23 |
| **NP_722519.2** | PHF13 | 1 | PHD finger protein 13 | 33.9 | 0.25 |
| **NP_077273.2** | PHF23 | 17 | PHD finger protein 23 | 33.9 | 0.32 |
| **NP_065940.1** | PHF12 | 17 | PHD finger protein 12 isoform 2 | 33.1 | 0.52 |
| **NP_001028733.1** | PHF12 | 17 | PHD finger protein 12 isoform 1 | 33.5 | 0.53 |
| **NP_001128627.1** | DPF1 | 19 | Zinc finger protein neuro-d4 isoform a | 29.6 | 5.8 |
| **NP_001128628.1** | DPF1 | 19 | Zinc finger protein neuro-d4 isoform c | 29.3 | 8.8 |
