# Supplementary material for: GSTCD and INTS12 Regulation and Expression in the Human Lung
Source: PLoS One. 2013 Sep 18;8(9):e74630. doi: 10.1371/journal.pone.0074630 (PMC3776747; doi:10.1371/journal.pone.0074630)
Supplement: Table S2 — A. 100 “resistant” smokers whole exome re-sequencing. An exome re-sequencing project (results unpublished) identified 1 non-synonymous SNP and 1 synonymous SNP in the coding regions of INTS12 which were listed in dbSNP v137 but which were not in the 1000 Genomes Project imputation reference panel. Both SNPs occurred as a singleton call (1 heterozygote, no ALT allele homozygotes) in the 100 individuals. rs146172950 is listed as a triallelic SNP in dbSNP137 with reference allele C and alternative allele T (frequency 0.049%) originally identified and submitted by the NHLBI Exome Sequencing Project and alternative allele A (frequency 0.016%) subsequently identified and submitted by the 1000 Genomes Project. The alternative allele T was observed in the 100 resistant smokers exome sequencing project. rs144650579 is listed in dbSNP137 with reference allele T and alternative allele C (frequency 0.085%) originally identified and submitted by the NHLBI Exome Sequencing Project. B. Targeted sequencing of GSTCD/INTS12 region. A targeted sequencing study (results unpublished) identified two intronic SNPs in GSTCD that do not appear in 1000 Genomes Project. All the SNPs identified in INTS12 appear in 1000 Genomes Project imputation reference panel. rs113987854 is listed in dbSNP137 with reference allele T and alternative allele A originally identified and submitted by Complete Genomics. No population frequency is available for this SNP (only counts based on one individual have been submitted to dbSNP137). (DOCX) [file pone.0074630.s006.docx]

**Table S2A. 100 “resistant” smokers whole exome re-sequencing.** An exome re-sequencing project (results unpublished) identified 1 non-synonymous SNP and 1 synonymous SNP in the coding regions of *INTS12* which were listed in dbSNP v137 but which were not in the 1000 Genomes Project imputation reference panel. Both SNPs occurred as a singleton call (1 heterozygote, no ALT allele homozygotes) in the 100 individuals. rs146172950 is listed as a triallelic SNP in dbSNP137 with reference allele C and alternative allele T (frequency 0.049%) originally identified and submitted by the NHLBI Exome Sequencing Project and alternative allele A (frequency 0.016%) subsequently identified and submitted by the 1000 Genomes Project. The alternative allele T was observed in the 100 resistant smokers exome sequencing project. rs144650579 is listed in dbSNP137 with reference allele T and alternative allele C (frequency 0.085%) originally identified and submitted by the NHLBI Exome Sequencing Project.

| rs | chr | pos | function | gene | REF | ALT | In 1000G? | In dbSNP? |
| --- | --- | --- | --- | --- | --- | --- | --- | --- |
| rs146172950 | 4 | 106604435 | Non-synonymous | *INTS12* | C | T | NO | YES |
| rs144650579 | 4 | 106621088 | Synonymous | *INTS12* | T | C | NO | YES |

**Table S2B. Targeted sequencing of *GSTCD*/*INTS12* region.** A targeted sequencing study (results unpublished) identified two intronic SNPs in *GSTCD* that do not appear in 1000 Genomes Project. All the SNPs identified in *INTS12* appear in 1000 Genomes Project imputation reference panel. rs113987854 is listed in dbSNP137 with reference allele T and alternative allele A originally identified and submitted by Complete Genomics. No population frequency is available for this SNP (only counts based on one individual have been submitted to dbSNP137).

| rs | chr | pos | function | gene | REF | ALT | In 1000G? | In dbSNP? |
| --- | --- | --- | --- | --- | --- | --- | --- | --- |
| rs113987854 | 4 | 106671050 | intronic | *GSTCD* | T | G | NO | YES |
| NA | 4 | 106679980 | intronic | *GSTCD* | C | T | NO | NO |

References:

1. Repapi, E., et al., *Genome-wide association study identifies five loci associated with lung function.* Nat Genet, 2010. **42**(1): p. 36-44.

2. Hancock, D.B., et al., *Meta-analyses of genome-wide association studies identify multiple loci associated with pulmonary function.* Nat Genet, 2010. **42**(1): p. 45-52.

3. Soler Artigas, M., et al., *Genome-wide association and large-scale follow up identifies 16 new loci influencing lung function.* Nat Genet, 2011. **43**(11): p. 1082-90.
