# Supplementary material for: GSTCD and INTS12 Regulation and Expression in the Human Lung
Source: PLoS One. 2013 Sep 18;8(9):e74630. doi: 10.1371/journal.pone.0074630 (PMC3776747; doi:10.1371/journal.pone.0074630)
Supplement: Table S3 — ChromStart: Start position in chromosome; ChromEnd: End position in chromosome; Name: name of transcription factor; Score: Score from 0-1000 (score for each peak, which reflects the posterior probability of the peak belonging to the irreproducible group). Search performed 30/01/2013. (DOCX) [file pone.0074630.s007.docx]

Table S3. Transcription Factor sites identified via the ENCODE project Chip-Seq assay in the region: chr4:106609150-106654787 (hg build37). ChromStart: Start position in chromosome; ChromEnd: End position in chromosome; Name: name of transcription factor; Score: Score from 0-1000 (score for each peak, which reflects the posterior probability of the peak belonging to the irreproducible group). Search performed 30/01/2013.

| **Chrom Start** | **Chrom End** | **Name** | **Score** | **Block Sizes** |
| --- | --- | --- | --- | --- |
| 106629675 | 106630143 | TAF1 | 1000 | 469 |
| 106629726 | 106630060 | GABP | 1000 | 335 |
| 106629741 | 106630002 | RXRA | 1000 | 262 |
| 106629750 | 106630012 | ELF1_(SC-631) | 1000 | 263 |
| 106629761 | 106629989 | Sin3Ak-20 | 1000 | 229 |
| 106629776 | 106629999 | BCLAF1_(M33-P5B11) | 1000 | 224 |
| 106629783 | 106629991 | PAX5-C20 | 940 | 209 |
| 106629652 | 106630104 | Pol2-4H8 | 909 | 453 |
| 106629673 | 106630035 | NFKB | 873 | 363 |
| 106629830 | 106629952 | IRF4_(M-17) | 873 | 123 |
| 106629768 | 106630047 | WHIP | 847 | 280 |
| 106629771 | 106630020 | HDAC2_(SC-6296) | 819 | 250 |
| 106629761 | 106630000 | Oct-2 | 817 | 240 |
| 106629761 | 106630000 | POU2F2 | 813 | 240 |
| 106629660 | 106629869 | ZBTB7A_(SC-34508) | 805 | 210 |
| 106629711 | 106630030 | Ini1 | 803 | 320 |
| 106629585 | 106630157 | Pol2 | 786 | 573 |
| 106629724 | 106630027 | p300 | 695 | 304 |
| 106629769 | 106629992 | ZEB1_(SC-25388) | 650 | 224 |
| 106629778 | 106629987 | SRF | 647 | 210 |
| 106629711 | 106630050 | GTF2F1_(RAP-74) | 631 | 340 |
| 106629791 | 106629974 | ETS1 | 618 | 184 |
| 106629725 | 106630088 | ELK4 | 617 | 364 |
| 106629761 | 106630024 | ZZZ3 | 611 | 264 |
| 106629749 | 106630012 | SP1 | 602 | 264 |
| 106629573 | 106629928 | Pol2(b) | 552 | 356 |
| 106629761 | 106630000 | BCL3 | 549 | 240 |
| 106629738 | 106629993 | MEF2A | 547 | 256 |
| 106629787 | 106630124 | YY1_(C-20) | 542 | 338 |
| 106630110 | 106630319 | ZBTB7A_(SC-34508) | 541 | 210 |
| 106631789 | 106632132 | GATA-2 | 533 | 344 |
| 106629752 | 106629995 | MEF2C_(SC-13268) | 511 | 244 |
| 106629771 | 106629986 | TCF12 | 508 | 216 |
| 106629749 | 106630004 | c-Myc | 503 | 256 |
| 106629751 | 106629986 | BCL11A | 502 | 236 |
| 106629752 | 106629967 | Egr-1 | 487 | 216 |
| 106629773 | 106630032 | PAX5-N19 | 469 | 260 |
| 106629771 | 106630005 | SIX5 | 447 | 235 |
| 106629749 | 106630044 | eGFP-JunD | 428 | 296 |
| 106629798 | 106629968 | YY1 | 401 | 171 |
| 106629788 | 106630071 | Pol2(phosphoS2) | 396 | 284 |
| 106629773 | 106630022 | Mxi1_(bHLH) | 383 | 250 |
| 106629749 | 106629988 | HNF4A_(H-171) | 369 | 240 |
| 106629737 | 106630055 | TBP | 347 | 319 |
| 106629799 | 106629974 | JunD | 337 | 176 |
| 106629759 | 106630048 | eGFP-JunB | 333 | 290 |
| 106629784 | 106629999 | BATF | 312 | 216 |
| 106629651 | 106630129 | HEY1 | 308 | 479 |
| 106629781 | 106629984 | Znf143_(16618-1-AP) | 268 | 204 |
| 106629739 | 106629998 | GATA3_(SC-268) | 251 | 260 |
| 106629576 | 106630025 | ZNF263 | 231 | 450 |
| 106629723 | 106630006 | GTF2B | 218 | 284 |
| 106629715 | 106630024 | TCF4 | 209 | 310 |
| 106629714 | 106629949 | FOSL2 | 202 | 236 |
| 106629783 | 106630042 | BRCA1_(C-1863) | 200 | 260 |
| 106629761 | 106630012 | PU.1 | 182 | 252 |
| 106629704 | 106630153 | GATA-1 | 181 | 450 |
| 106629753 | 106630013 | USF-1 | 180 | 261 |
| 106629782 | 106630065 | IRF1 | 180 | 284 |
| 106629728 | 106630011 | STAT3 | 175 | 284 |
| 106629765 | 106629988 | TAF7_(SQ-8) | 175 | 224 |
| 106629796 | 106630031 | Max | 173 | 236 |
| 106629745 | 106629944 | E2F6_(H-50) | 159 | 200 |
| 106629808 | 106630147 | E2F4 | 146 | 340 |
| 106629837 | 106630032 | Pbx3 | 144 | 196 |
| 106629715 | 106630058 | GATA-2 | 143 | 344 |
| 106629744 | 106630003 | FOXA1_(C-20) | 134 | 260 |
| 106629749 | 106630018 | GR | 124 | 270 |
| 106629769 | 106630143 | NRSF | 114 | 375 |
| 106629703 | 106630062 | STAT1 | 111 | 360 |
| 106629771 | 106629994 | FOXA2_(SC-6554) | 106 | 224 |
| 106629759 | 106630034 | RFX5_(N-494) | 86 | 276 |
| 106629781 | 106629990 | Rad21 | 78 | 210 |
| 106628944 | 106629193 | USF-1 | 77 | 250 |
| 106629691 | 106629940 | TAL1_(SC-12984) | 54 | 250 |
| 106629760 | 106629979 | USF1_(SC-8983) | 43 | 220 |
| 106629701 | 106630012 | CHD2_(N-1250) | 42 | 312 |
