# Supplementary material for: GSTCD and INTS12 Regulation and Expression in the Human Lung
Source: PLoS One. 2013 Sep 18;8(9):e74630. doi: 10.1371/journal.pone.0074630 (PMC3776747; doi:10.1371/journal.pone.0074630)
Supplement: Table S5 — Pre: pre-bronchodilator; Post: post-bronchodilator; Laval (Laval University, Canada), Groningen (University of Groningen, The Netherlands) and UBC (University of British Columbia, Canada) refer to the cohorts recruited from each centre. Data are presented as mean ± standard deviation. (DOCX) [file pone.0074630.s009.docx]

**Table S5.** **Clinical characteristics of 848 individuals participating in the eQTL study.** Pre: pre­bronchodilator; Post: post-bronchodilator; Laval (Laval University, Canada), Groningen (University of Groningen, The Netherlands) and UBC (University of British Columbia, Canada) refer to the cohorts recruited from each centre. Data are presented as mean ± standard deviation.

| **Characteristics** | Laval (n=403) | UBC (n=270) | Groningen (n=175) |
| --- | --- | --- | --- |
| Age (years) | 63.4 ± 9.8 | 63.9 ± 10.0 | 59.7 ± 10.0 |
| Male/female (n) | 224/179 | 144/126 | 92/83 |
| BMI (kg/m²) | 26.6 ± 5.2 | 25.7 ± 5.4 | 24.6 ± 4.1 |
| Percent predicted FEV_1_ (pre) | 80.54 ± 19.02 | 79.69 ± 22.65 | 72.36 ± 24.85 |
| FEV_1_/FVC (pre) | 0.67 ± 0.10 | 0.68 ± 0.12 | 0.64 ± 0.16 |
| Pack years (n) | 48.6 ± 27.4 | 45.9 ± 28.9 | 34.9 ± 16.2 |
| Smoker (n,%) | 89 (22.1) | 91 (35.3) | 43 (25.0) |
| Ex-Smoker (n,%) | 281 (69.7) | 150 (58.6) | 117 (68.0) |
| Non-Smoker (n,%) | 33 (8.2) | 16 (6.1) | 12 (7.0) |
