# Supplementary material for: GSTCD and INTS12 Regulation and Expression in the Human Lung
Source: PLoS One. 2013 Sep 18;8(9):e74630. doi: 10.1371/journal.pone.0074630 (PMC3776747; doi:10.1371/journal.pone.0074630)
Supplement: Table S6 — logFC: log-fold difference between 2 experimental groups; Average expression is between all samples; t: t-statistic describing differential expression; P Value: Unadjusted P value; Adjusted p value controls for false discovery rate; Beta-coefficient: log-odds ratio. n/s: no significance observed. Significant effects are shown in bold and italicised. (DOCX) [file pone.0074630.s010.docx]

**Table S6. Fetal lung gene array data comparing *GSTCD* and *INTS12* expression during pseudoglandular and canalicular stages of lung development.** logFC: log-fold difference between 2 experimental groups; Average expression is between all samples; t: t-statistic describing differential expression; *P* Value: Unadjusted *P* value; Adjusted p value controls for false discovery rate; Beta-coefficient: log-odds ratio. n/s: no significance observed. Significant effects are shown in bold and italicised.

| **Gene Entrez ID** | **Probe ID** | **logFC** | **Average Expression** | **t** | ***P* Value** | **Adjusted *P* Value** | **Beta- coefficient** | **Significant Effect** |
| --- | --- | --- | --- | --- | --- | --- | --- | --- |
| ***GSTCD*** | 220063_at | -0.0037 | 5.6144 | -2.3059 | 0.0265 | 0.0862 | -0.0037 | n/s |
| ***GSTCD*** | 1554518_at | -0.0055 | 5.8000 | -3.2205 | 0.0026 | ***0.0150*** | -0.0055 | Decreased expression with age |
| NA | 241126_at | 0.0012 | 3.4619 | 1.0242 | 0.3120 | 0.4878 | 0.0012 | n/s |
| ***GSTCD*** | 235387_at | -0.0115 | 6.3236 | -4.4776 | 0.0001 | ***0.0009*** | -0.0115 | Decreased expression with age |
| ***INTS12*** | 218616_at | -0.0040 | 8.2248 | -1.7318 | 0.0911 | 0.2096 | -0.0040 | n/s |
